# Vibration-Based Numerical and Experimental Analysis on Delamination Damage Identification for Composite Laminates

**DOI:** 10.3390/ma19194146

**Published:** 2026-09-29

**Authors:** Shuxia Tian, Guangke Li, Shuai Liu

**Affiliations:** 1 Henan Provincial Key Laboratory of Intelligent Manufacturing of High-End Equipment, Zhengzhou University of Light Industry, Zhengzhou 450001, China; ligk@zzuli.edu.cn; 2 Henan Huamai Communication Technology Limited Liability Company, Zhengzhou 450003, China; liushuai2662@163.com

**Keywords:** composite laminates, delamination damage, modal shape curvature, uniform load surface curvature, baseline-free damage identification, structural health monitoring

## Abstract

Composite laminates possess excellent specific mechanical performance and are widely adopted in aerospace, rail transit and national defense industries, whereas interlayer delamination, as a typical concealed damage, seriously threatens structural safety. Most existing vibration-based delamination detection algorithms rely on intact structure baseline data, which restricts their practical engineering application. Aiming at this limitation, this paper investigates two baseline-free delamination identification approaches combining modal shape curvature and uniform load surface (ULS) curvature for eight-layer symmetric cross-ply composite laminates. Finite-element models containing boundary-layer and middle-layer delamination are established using the node-merging method. Modal shape curvature and ULS curvature are computed via the central difference method, and two baseline-free schemes, namely the surface smoothing algorithm and curvature mode change rate method, are implemented for damage feature extraction. Three quantitative evaluation indices, including Peak to Background Ratio (PBR), localization error and Signal to Noise Ratio (SNR), are introduced to objectively compare the identification performance of different curvature indicators and algorithms. Numerical results demonstrate that both methods can effectively locate delamination under 3% random measurement noise. Boundary-layer delamination yields more prominent damage features than middle-layer delamination; modal shape curvature outperforms ULS curvature with 1.29–1.53 times higher PBR and 1.23–1.52 times higher SNR, alongside reduced localization error. Under identical conditions, the curvature mode change rate method achieves 1.51–1.99 times higher PBR, 1.22–1.36 times higher SNR and localization error reduced to 0.50–0.79 of that obtained by the surface smoothing algorithm. Scanning laser Doppler vibration tests on carbon-fiber specimens with preset delamination defects further validate the proposed methods. Experimental results reveal that under actual measurement noise, the curvature mode change rate method attains maximum PBR of 12.56, localization error down to 6.46 mm and SNR up to 14.13 dB, exhibiting better comprehensive performance compared with the surface smoothing algorithm. This work provides quantitative reference for baseline-free delamination detection of composite laminates and offers a feasible technical route for practical non-destructive inspection.

## 1. Introduction

In recent decades, driven by rapid economic growth, significant advances have been made in advanced manufacturing, machining and material fabrication technologies. Composite laminates, as a representative and essential structural form of composite materials, are characterized by light weight and multifunctional integration. Owing to their exceptional mechanical attributes—high specific strength, high specific stiffness, favorable impact resistance, and superior sound insulation and vibration damping capacity—composite laminates have been widely deployed in a broad range of high-end and high-technology sectors, including aerospace, high-speed railways, national defense, chemical textiles and marine engineering [1,2,3].

However, defects are inevitably introduced into laminate structures during fabrication and long-term service. Adverse factors such as imperfect manufacturing procedures, external impact loads, and cyclic service loads readily initiate structural damage, among which delamination represents the most common and typical failure mode [4,5,6]. Delamination substantially degrades the mechanical performance and operational reliability of composite laminate components. When delamination arises in critical load-bearing structural members, local structural failure may be triggered, potentially resulting in catastrophic accidents. Accordingly, accurate and efficient identification of delamination damage in composite laminates carries vital engineering significance and research value.

Currently, diversified damage identification technologies have been developed for composite material structures. Among them, vibration characteristic-based damage detection methods have gained widespread attention and engineering application due to their advantages of non-destructiveness, simple operation, and strong adaptability [7,8]. The mainstream vibration-based technical approaches for composite damage localization mainly include four categories: vibration mode characteristic variation method [9,10], modal curvature distribution method [11,12], flexibility matrix reconstruction method [13], and strain energy distribution analysis method [14].

Numerous existing studies have verified that modal curvature is an extremely sensitive characteristic indicator for characterizing local structural stiffness degradation and micro-damage, which shows unique superiority in local damage localization of composite laminates. To enhance the resolution performance of vibration-based damage identification for composite laminates, Chen et al. [15] developed a vibration-based damage detection framework with tunable resolution, which offers new insights for improving curvature-based damage feature extraction under complex composite service conditions. Shao et al. [16] conducted targeted investigations into interfacial bonding damage of composite materials, further extending the application scope of vibration modal-based damage detection to composite joint interfaces. Sun et al. [17] explored the fundamental theories of modal feature-driven structural health monitoring and revealed the response mechanism of curvature-related modal characteristics to local structural defects under multi-source excitations.

Apart from global vibration modal technologies, ultrasonic guided-wave methods have attracted growing interest in composite laminate delamination monitoring, benefiting from long propagation distance and high sensitivity toward subsurface inter-ply defects; nevertheless, guided-wave approaches usually require dense piezoelectric sensor networks and suffer from complex wave dispersion and mode conversion in anisotropic composite media [18,19]. In recent years, machine-learning and deep-learning-driven structural-health-monitoring schemes have also been rapidly developed. These data-driven methods can automatically extract damage features from vibration or guided-wave signals and realize intelligent damage localization and severity evaluation; yet, their performance heavily relies on large-scale labeled training datasets, which are often difficult to acquire for real composite engineering components [3,20].

Despite substantial theoretical and methodological advances in existing research, most enhanced curvature-based damage detection approaches are still plagued by intrinsic limitations. A large proportion of these algorithms heavily rely on complete baseline data of the intact structure to perform differential analysis, which considerably constrains their practicability in real-world engineering applications where undamaged reference datasets are hardly accessible. Furthermore, existing investigations predominantly employ a single modal curvature metric for damage quantification, without carrying out systematic comparative evaluations among diverse curvature-derived features. Such practices result in unsatisfactory detection performance toward micro-scale delamination, while the noise-resistant robustness of these algorithms under complex in-service disturbances remains in urgent need of enhancement. Considering these unresolved research gaps and technical bottlenecks, there is a pressing need to develop innovative curvature-based identification algorithms free from baseline-dependent parameters, alongside comparative validations, to realize more precise and trustworthy delamination detection for composite laminates.

Based on the above research background and in response to the technical shortcomings of existing curvature-based damage identification methods, this paper takes eight-layer symmetric cross-ply composite laminates as the research object. Although modal curvature, uniform load surface (ULS) curvature and the surface smoothing technique have been reported in the existing literature, most prior studies rarely systematically compare the performance of these two curvature indicators together with two baseline-free identification algorithms for composite laminate delamination, and the influences of delamination depth (boundary-layer versus middle-layer delamination) on identification performance lack sufficient comparative investigation; moreover, relevant experimental verification under measurement noise interference for composite laminate specimens remains limited. Firstly, a finite-element numerical model based on the node-merging method was established to simulate composite laminates with various delamination damage conditions (including boundary-layer and middle-layer delamination) and intact states [21]. Modal analysis was performed to acquire structural vibration characteristic parameters, and the modal shape curvature and uniform load surface (ULS) curvature corresponding to damaged and undamaged structures were calculated subsequently. Based on previous research on these two curvature parameters [22,23,24], two improved baseline-free algorithms, namely the surface smoothing algorithm and the curvature mode change rate method, are implemented for delamination damage identification of composite laminates. This work specifically focuses on: (1) comparative analysis of damage identification performance between modal shape curvature and ULS curvature indicators; (2) investigation of the effect of delamination depth (boundary-layer and middle-layer delamination) on detection results; (3) comparative evaluation of the surface smoothing algorithm and curvature mode change rate method for laminate delamination; and (4) experimental validation using laser Doppler vibration tests on carbon-fiber composite specimens with preset delamination defects under noise interference. Finally, the effectiveness and anti-noise performance of the above two baseline-free identification approaches is demonstrated by processing experimental vibration modal data from damaged laminates.

## 2. Numerical Method for Damage Detection of Laminated Plates

Once damage occurs in engineering structures, it will cause changes in their characteristic parameters (mass, stiffness, damping, etc.), which will lead to changes in modal information (modal frequency, modal shape, modal damping, modal flexibility, etc.)—that is, the damage information of the structure is reflected in the change in its modal information. In practical engineering, it is difficult to identify minor damage by using structural modal information directly because minor damage has little influence on structural dynamic response parameters and is greatly disturbed by external noise. Therefore, in order to optimize the detection effect, curvature mode is selected as a sensitive parameter for damage detection.

### 2.1. Modal Curvature Calculation Algorithms

#### 2.1.1. Uniform Load Surface (ULS)

Based on the flexibility matrix, numerous researchers adopt uniform load surface (ULS) values for structural damage detection [25,26]. In this paper, the uniform load surface (ULS), a derivative of modal flexibility, is constructed from the vibration parameters acquired through numerical simulation or experimental measurement as follows:

(1) $$u\left(i\right)=\bar{f}\left(i\right)=\sum_{j=1}^{n}f_{i,j}=\sum_{j=1}^{n}\left(\sum_{k=1}^{m}\frac{\phi^{k}\left(i\right)\phi^{k}\left(j\right)}{\omega_{k}^{2}}\right)=\sum_{k=1}^{m}\frac{\phi^{k}\left(i\right)\sum_{j=1}^{n}\phi^{k}\left(j\right)}{\omega_{k}^{2}}$$

where $\left\{\bar{f}\right\}=F\cdot L,\;L={\left\{1,\;1,\;\ldots ,\;1\right\}}_{1\times n}^{T}$ denotes a column vector with all elements equal to unity. Physically, this vector represents the application of unit magnitude loads simultaneously at all measurement points over the plate for the derivation of the uniform load surface. **F** denotes the flexibility matrix. The term $f_{i,j}$ represents the flexibility coefficient at the *i*-th point induced by a unit load applied at the *j*-th point. $\phi^{k}\left(i\right)$ corresponds to the entry of the *k*-th unit mass-normalized mode shape at the *i*-th measurement point. $\omega_{k}^{2}$ stands for the squared angular frequency of the *k*-th mode. Herein, *m* is the count of modes extracted from experimental measurements, and *n* denotes the total number of physical measurement locations for testing.

Equation (1) reveals that the contribution of each mode to modal flexibility is inversely proportional to the square of its corresponding natural frequency. Owing to this property, modal flexibility exhibits rapid convergence with an increasing number of included modes. Consequently, a small set of lower-order modes can well approximate the full flexibility matrix.

#### 2.1.2. Modal Curvature Calculation Based on Central Difference Method

In this paper, modal curvature and ULS curvature parameters are used as damage indicators to study the delamination damage of laminates.

Modal curvature is derived from modal shape. Modal shape is a basic attribute parameter of structure, which covers the vibration information of structure at different positions. It contains information related to the location and degree of the damage. The modal shapes can be used to locate damage well due to their rich damage information, but they are less sensitive to minor damage. Studies have shown that the local stiffness of the damaged position will decrease after the structure is damaged, which will cause the curvature of the damaged position to increase. Thus, the curvature is closely related to the location of the damage, i.e., the curvature modal method. In this paper, the central difference method was used to calculate the modal shape curvature and ULS curvature as follows:

(2) $$\begin{array}{c} u_{xx}\left(x_{i},y_{j}\right)=\frac{u\left(x_{i+1},y_{j}\right)-2u\left(x_{i},y_{j}\right)+u\left(x_{i-1},y_{j}\right)}{h_{x}^{2}}\\ u_{yy}\left(x_{i},y_{j}\right)=\frac{u\left(x_{i},y_{j+1}\right)-2u\left(x_{i},y_{j}\right)+u\left(x_{i},y_{j-1}\right)}{h_{y}^{2}} \end{array}\;$$

where *u*(*x_i_*, *y_j_*) is the modal value or ULS value at point (*x_i_*, *y_j_*), *u_xx_*(*x_i_*, *y_j_*) and *u_yy_*(*x_i_*, *y_j_*) are the second-order partial differentials along the *X* or *Y* direction, and *h_x_* and *h_y_* are the uniform grid spacings in the corresponding directions.

### 2.2. Damage Detection Algorithms Based on Surface Smoothing Method

Damage detection approaches relying on baseline or pristine data often suffer from limited reliability in practical implementations, as a priori measurements acquired under healthy conditions are highly susceptible to environmental interference. To avoid this difficulty, Wu et al. [27,28] proposed the “Gapped-smoothing” technique to detect damages in beam and plate structures respectively. The basic idea of the method is that the ULS curvature of the sandwich plate, without any damage, has a smooth surface. The surface smoothing method can eliminate the peaks hiding in the ULS curvature caused by delamination damage. Therefore, the approximate ULS curvature without delamination damage can be established for the difference method.

Based on the surface smoothing method, the smoothed ULS curvature at each point (*x_i_*, *y_j_*) can be approximated by the following formula:

(3) $$u_{fitting}\left(x,y\right)=\sum_{i=0}^{2}\sum_{j=0}^{2}C_{ij}x^{i}y^{j}$$

where the coefficient *C_ij_* for an approximate curvature value without delamination defect can be evaluated by a curve-fitting process. Particularly, to obtain the smoothed ULS curvature at point (*x_i_*, *y_j_*), curvature data at all the adjacent points, but not the point (*x_i_*, *y_j_*) itself, are used to evaluate the coefficient *C_ij_* in Equation (3). This process is repeated for each measuring point to give a smooth curvature value to model the undamaged laminates.

In this study, a local 3 × 3 gapped fitting window is applied for each interior measuring point (*x_i_*, *y_j_*). For the target point to be reconstructed, the point itself is excluded from fitting inputs following the gapped-smoothing principle. The surrounding eight neighboring nodes within the 3 × 3 stencil are selected as sampling data. A second-order two-dimensional polynomial is utilized to fit the local curvature surface and solve the fitting coefficients in Equation (3), so as to obtain the approximated undamaged curvature value at the target coordinate.

Special treatment is required for boundary and corner measuring points, where a complete set of eight neighboring points cannot be acquired due to plate geometric limits. For these boundary locations, the fitting window is adaptively shrunk according to the number of physically available adjacent measuring points. The identical second-order two-dimensional polynomial fitting procedure is carried out using only the valid surrounding points. The reconstructed undamaged curvature values for boundary points are then obtained accordingly.

The presence of the peak in the ULS curvature due to delamination defect can then be detected by subtracting the smoothed curvature from the estimated curvature of the damaged structure. The damage index map is given as:

(4) $$d\left(x_{i},y_{j}\right)={\left(u_{measured}(x,y)-u_{fitting}(x,y)\right)}^{2}$$

For an undamaged structure, the difference between the above two sets of measured ULS curvature is only due to the measurement noise. Therefore, values of the damage index map, $d\left(x_{i},y_{j}\right)$, slightly oscillate around zero without any distinct peak. In contrast, peaks or slopes will clearly show up at the damaged zone of the plate if the structure is damaged.

### 2.3. Damage Detection Algorithms Based on Curvature Mode Change Rate

Similar to the surface smoothing method, the curvature mode change rate method does not require parameters obtained from the initially intact structure. Cheng et al. [29] have used the first four orders of curvature mode difference mean index and the curvature index of the modal flexibility change rate to accurately locate the damage to the ship’s beam structure. In this paper, the detection method based on curvature mode change rate was adopted for the delamination defect identification for cantilevered composite laminates by using the ULS curvature values.

The basic idea of this method is that the local stiffness degradation caused by delamination will be expressed by the volatility of change rate of curvature. For two-dimensional plate structures, curvature abruptness may occur along either the *X* direction or *Y* direction over the measurement grid. Therefore, curvature mode change rates are computed separately along the *X* and *Y* spatial directions.

For a uniform grid with grid spacing $h_{x}$ along *X*-axis and $h_{y}$ along *Y*-axis:

The curvature mode change rate along the *X* direction at node $\left(i,j\right)$ for the *k*-th mode is defined as:

(5) $$\begin{array}{c} R_{C,X,k}\left(i,j\right)=\frac{u_{xx,k}\left(i+1,j\right)-u_{xx,k}\left(i,j\right)}{h_{x}} \end{array}$$

The curvature mode change rate along the *Y* direction at node $\left(i,j\right)$ for the *k*-th mode is defined as:

(6) $$\begin{array}{c} R_{C,Y,k}\left(i,j\right)=\frac{u_{yy,k}\left(i,j+1\right)-u_{yy,k}\left(i,j\right)}{h_{y}} \end{array}$$

where $u_{xx-k}$, $u_{yy,k}$ denotes either modal shape curvature or ULS curvature of the *k*-th mode, calculated by Equation (2); $h_{x}$ and $h_{y}$ are uniform grid spacings in *X* and *Y* directions, respectively.

For multi-mode superposition adopted in this work, the curvature mode change rate components of the first three modes are normalized and summed for each direction.

To construct the final two-dimensional damage map, the Euclidean norm is applied to fuse the two directional components at each grid point $\left(i,j\right)$:

(7) $$\begin{array}{c} R_{C}\left(i,j\right)=\sqrt{{\left(\sum_{k=1}^{3}R_{C,X,k}\left(i,j\right)\right)}^{2}+{\left(\sum_{k=1}^{3}R_{C,Y,k}\left(i,j\right)\right)}^{2}} \end{array}$$

The fused index $R_{C}\left(i,j\right)\;$ serves as the final damage index for plotting 2D damage maps. This combination strategy simultaneously captures abnormal curvature features originating from both *X* and *Y* direction spatial variations, and the magnitude peaks of $R_{C}\left(i,j\right)$ correspond to potential delamination locations. For the delamination identification of composite laminates, defect information is extracted from the spatial peak distribution of the fused curvature mode change rate field.

### 2.4. Quantitative Performance Evaluation Metrics

Simply comparing the absolute magnitude of damage indices is not rigorous for performance comparison, because different curvature-derived damage indicators possess distinct physical definitions, dimensions and inherent numerical scales. In this study, three quantitative metrics, Peak to Background Ratio (PBR), localization error, and Signal to Noise Ratio (SNR), are introduced to objectively evaluate the delamination identification performance of different curvature indicators and baseline-free algorithms.

The Peak to Background Ratio (PBR) quantifies how significantly the damage feature stands out against the undamaged background field, which eliminates the influence of intrinsic numerical-scale differences among various damage indexes:

(8) $$\begin{array}{c} PBR=\frac{D_{peak,damage}}{{\bar{D}}_{background}} \end{array}$$

where $D_{peak,damage}$ is the maximum amplitude of damage index extracted within the spatial range of the true preset delamination region; ${\bar{D}}_{background}$ denotes the mean value of damage index calculated over all undamaged measurement points on the laminate. A larger PBR value means the damage signature is more distinguishable from the background fluctuation. For cases with multiple delamination damages, all damage-related peaks are extracted from the curvature map, and the minimum peak value is adopted to compute PBR, which evaluates the feature saliency of the least detectable damage.

Localization error $E_{loc}$ evaluates the spatial positioning accuracy of the detected delamination. It is computed as the Euclidean distance between the detected damage-peak coordinate $\left(x_{detect},y_{detect}\right)$ and the true geometric center coordinate $\left(x_{true},y_{true}\right)$ of the predefined delamination:

(9) $$\begin{array}{c} E_{loc}=\sqrt{{\left(x_{detect}-x_{true}\right)}^{2}+{\left(y_{detect}-y_{true}\right)}^{2}} \end{array}$$

$E_{loc}$ is given in millimeters. Smaller localization error indicates higher spatial identification accuracy. For cases with multiple delamination damages, the distance between predicted position and true delamination coordinate is calculated for each damage. The average value over all damage sites is taken as the localization error for this case.

The Signal to Noise Ratio (SNR) reflects the anti-interference capability of damage identification indicators:

(10) $$\begin{array}{c} SNR=20\log_{10}\left(\frac{D_{peak,damage}}{\sigma_{background}}\right) \end{array}$$

where $\sigma_{background}$ represents the standard deviation of damage index values sampled over undamaged background zones. Higher SNR corresponds to a stronger damage-related signal relative to background disturbances. For cases with multiple delamination damages, SNR is solved uniformly based on the curvature signal of the entire field. The signal region covers all multiple delamination zones, while noise is sampled from intact background regions far from damage. A single SNR value is obtained for each case to quantify the overall noise robustness of the curvature indicator.

## 3. Numerical Examples

### 3.1. Finite-Element Model

The composite laminate specimen is a carbon-fiber/epoxy resin reinforced polymer (CFRP) composite, which takes continuous carbon fiber as the reinforcing phase and high-strength epoxy resin as the bonding matrix. The epoxy resin matrix tightly bonds adjacent fiber layers, undertakes interfacial load transfer, and maintains the overall structural integrity of the laminate, which is a standard and widely applied composite material system in aerospace and rail transit engineering.

In this work, eight-ply symmetric cross-ply composite laminates with a stacking sequence of [0°/90°] were modeled. The overall structural dimensions of the laminate are 400 mm × 260 mm × 1.6 mm, with a single ply thickness of 0.2 mm and an overall density of 1566 kg/m^3^. The key mechanical properties of the composite are set as follows: *E*_1_ = 172.7 GPa, *E*_2_ = E_3_ = 7.2 GPa, *µ*_12_ = *µ*_13_ = 0.3, *G*_12_ = *G*_13_ = 3.76 GPa, and *G*_23_ = 2.8 GPa.

All mechanical parameters used in the finite-element models are experimentally calibrated values obtained from tensile and shear tests of actual composite specimens, rather than hypothetical numerical parameters, which can ensure the consistency between numerical simulation and experimental test conditions.

Numerical simulations were performed within ANSYS Mechanical, version 19.2. The composite plies were discretized using SHELL181 layered shell elements, which are four-node linear shell elements suitable for thin to moderately thick composite laminates and support layered section definition for individual ply properties and orientation. Each physical ply was explicitly defined within the shell section command, assigning corresponding ply thickness and fiber orientation angle for each of the eight plies.

The global uniform in-plane mesh size was set to 20 mm × 20 mm; the model consisted of 260 elements and 294 nodes in total. This mesh configuration was validated to adequately capture the global vibration characteristics of the laminate. The left edge of the plate was defined as the fixed boundary, where all translational and rotational degrees of freedom were fully constrained.

In practical service scenarios, composite laminates often consist of multiple material constituents. Inter-ply interfaces act as weak locations where delamination damage readily initiates. Delamination is one of the dominant failure modes for laminated composites. A well-defined delamination damage model is a prerequisite for investigating delamination failure in composite laminates. Delamination refers to inter-ply delamination between adjacent plies in a laminated structure. Damaged laminates exhibit complex mechanical responses, which introduce substantial challenges for numerical analysis and complicate the accurate characterization of geometric and mechanical features of delamination defects.

Generally, analytical models and numerical models are the two mainstream categories for delamination damage analysis of composite laminates. This study adopts finite-element numerical simulation for laminate damage analysis; thus, only the establishment of the numerical model is elaborated on in detail. In this paper, the node-merging method is employed to simulate laminate delamination damage [30]. Specifically, the start and end nodes of the delamination region are merged, while the intermediate nodes within the delamination area remain unmerged. The detailed implementation of this method is illustrated in Figure 1, where *m* represents the grid node number and *n* denotes the element number.

To prevent unphysical interpenetration (surface penetration) between the freed upper and lower delaminated surfaces during modal computation, contact pairs were defined over all unmerged delamination interfaces with contact type “no-separation” disabled and pure penalty contact algorithm activated. Contact behavior was set as frictionless; only compressive normal contact force is permitted, while tensile separation is allowed. This contact setup prohibits element surface penetration without introducing artificial bonding stiffness within the delaminated area.

To simulate typical hidden delamination defects under real-service conditions and further investigate the influence of delamination depth on damage identification performance, two representative delamination locations at different through-thickness positions are designed for the eight-ply laminate structure: boundary-layer delamination between the first and second plies near the laminate surface, and middle-layer delamination between the fourth and fifth plies at the mid-thickness of the laminate. The schematic diagram of delamination damage at different depths is shown in Figure 2.

To verify the feasibility of the above-mentioned detection methods, damage cases containing two or three delamination defects at different positions are adopted as calculation models for numerical analysis, as illustrated in Figure 3. Figure 3a corresponds to the model with two delamination defects, while Figure 3b corresponds to the model with three delamination defects. A fixed-end boundary condition was applied along the left edge of the laminate. All translational and rotational degrees of freedom along this edge were fully constrained in the finite-element model.

### 3.2. Structural Modal Analysis

Based on the above damage and intact models, modal analysis of the structures was carried out by using finite-element software ANSYS. The first six modal frequencies of the above laminated plates are shown in Table 1. It can be observed that the overall natural frequencies decrease once delamination damage is introduced into the structure. The corresponding vibration modes of each frequency in Table 1 are common vibration modes of cantilever plates, mainly including out-of-plane bending and torsional deformation. There is no significant difference that can be directly identified between the vibration modes of intact and damaged models, and the weak features caused by damage can only be identified by the signal processing methods proposed later in this article.

It should be noted that modal curvature, as the second-order spatial derivative of modal shape, is theoretically sensitive to spatial discretization. Formal multi-density mesh convergence calculations were not performed in this work. Nevertheless, the 20 mm × 20 mm mesh adopted here has proven sufficient from our accumulated experience on composite laminate vibration simulation. At the same time, in Section 4.3 of this article, the simulated and measured natural frequencies and vibration modes are in good agreement, indirectly proving that the discrete error has been effectively controlled. This study focuses on relative comparison of damage identification performance among different indicators and algorithms instead of precise absolute quantification of damage index magnitudes. Further dedicated mesh convergence study will be carried out in future quantitative investigations.

### 3.3. Damage Identification Results

#### 3.3.1. Calculation of Modal Shape Curvature and ULS Curvature

Two kinds of curvature parameters, i.e., modal shape curvature and ULS curvature, are employed to construct damage identification indicators, and the damage identification results obtained from these two curvature parameters were compared in this study.

For modal shape curvature analysis, superposition of multiple low-order modes can integrate multi-order damage-sensitive information and reduce the risk that identification results are dominated by the peculiar local deformation feature of a single-order mode. Therefore, to evaluate the influence of adopted mode number on damage identification performance, preliminary numerical tests were carried out by comparing identification results using the first order, first–second order, and first–third order superimposed modal shapes. It is observed that using only the first-order mode provides limited damage feature amplitude. Superposition of the first two modes improves the visibility of damage peaks. Further inclusion of the third-order mode yields well-defined, stable damage peaks without introducing obvious spurious noise peaks. Adding higher-order modes (above the third order) brings little improvement to identification quality but amplifies numerical noise. Therefore, this article ultimately chooses to normalize the first three modal shapes and superimpose them to complete the calculation of modal shape curvature.

It should be noted that the uniform load surface (ULS) is derived from modal flexibility. According to the flexibility theory, the contribution of each mode to modal flexibility is inversely proportional to the square of natural frequency; hence, lower-order modes dominate the flexibility reconstruction. Therefore, to maintain parameter consistency, the first three modal parameters consistent with those used for modal shape curvature calculation are selected in this work. The modal shapes are normalized first, followed by the calculation of ULS using Equation (1), and the corresponding curvature values are further obtained via the central difference method (Equation (2)). This ensures a fair comparison between the two curvature indicators.

It should be noted that, considering the influence of actual testing noise, this article uniformly adds 3% random noise to the normalized first three modal data, and then calculates the subsequent modal shape curvature and ULS curvature separately.

Based on the above-mentioned curvature parameters, two damage detection algorithms, namely the surface smoothing algorithm and the curvature mode change rate method, were adopted to identify delamination damage in laminates with various defect configurations. The identification outcomes obtained from these two algorithms are presented and compared in the following subsections.

#### 3.3.2. Damage Identification Results of Surface Smoothing Method

The surface smoothing algorithm is adopted to process the modal shape curvature and ULS curvature with 3% random noise for each damage model of the laminate. The cusps appearing at the damage were eliminated, and the corresponding modal parameters of the corresponding undamaged laminates were obtained. Then, the approximated undamaged model was distinguished from the damage model parameters, and the damage identification index was established by using Equation (4) to complete the damage identification of the laminate. The damage identification results are shown in Figure 4 and Figure 5. The *X* and *Y* axes in the figures represent the length and width of the plate, and the *Z* axis is the damage index *d*.

From Figure 4 and Figure 5, it can be seen that when delamination damage occurs in the cantilever laminated board, the surface smoothing algorithm can effectively identify various delamination defects under 3% random noise interference in the modal shape. Numerical results reveal that the value of damage identification index increases for damage located closer to the fixed-end boundary. Moreover, delamination depth exerts a notable influence on the magnitude of damage index for defects at the same in-plane position: boundary-layer delamination between the first and second plies yields a higher damage index compared with middle-layer delamination between the fourth and fifth plies, demonstrating that near-surface delamination produces more prominent damage features than mid-thickness delamination.

#### 3.3.3. Damage Identification Results of Curvature Mode Change Rate Method

Curvature mode change rate method was derived from curvature mode. Different from the surface smoothing algorithm, it did not need to make a difference between approximate undamaged model and damage model parameters. The curvature mode change rate method can directly identify damage by using the change rates of modal shape curvature and ULS curvature with 3% random noise. The damage identification results are shown in Figure 6 and Figure 7. The X and Y axes in the figures represent the length and width of the plate. Z axis is the change rate of the curvature mode, and the peak value will appear at the damage point, which is used to characterize the delamination damage of laminates.

As can be seen from Figure 6 and Figure 7, under the interference of 3% random noise, the curvature mode change rate method can effectively identify the approximate locations and number of damages in the laminate without relying on the parameters of the original undamaged model. Meanwhile, the phenomenon by which boundary-layer delamination produces more prominent damage features than middle-layer delamination is also observed, and these conclusions are consistent with those obtained by the surface smoothing algorithm. It should be emphasized that the conclusion that boundary-layer delamination is easier to detect than middle-layer delamination is derived from numerical simulation only, as specimens with middle-layer delamination were not manufactured for experimental testing in this study.

However, two parameters, namely ULS curvature and modal shape curvature, and two methods are adopted to construct damage identification indicators in this study. Further quantitative analysis is required to compare the advantages and disadvantages of the curvature parameters or identification methods.

### 3.4. Quantitative Performance Evaluation

Although the location and quantity of delamination damage can be intuitively identified from Figure 4, Figure 5, Figure 6 and Figure 7, two curvature values with distinct physical definitions and two different damage index construction methods are adopted in this study. To avoid misleading performance comparisons caused solely by the absolute amplitudes of damage indices that differ in physical definition, dimension and intrinsic numerical scale, three quantitative metrics, i.e., Peak to Background Ratio (PBR), localization error *E*_loc_, and Signal to Noise Ratio (SNR), are introduced to objectively evaluate the delamination identification performance of different curvature indicators and baseline-free algorithms.

The damage index values calculated by the surface smoothing algorithm and curvature mode change rate method were extracted for both the ULS curvature-based and modal shape curvature-based schemes. These values were substituted into Equations (8)–(10) to compute the PBR, *E_loc_* and SNR for various damage cases under the two identification methods. The calculation results are summarized in Table 2 and Table 3.

As can be seen from Table 2 and Table 3, PBRs obtained by the two methods are greater than 10 for all cases except the three-damage cases in Table 2. The relatively low PBRs in these special cases arises from the low amplitude of damage responses for damages far from the fixed-end constraint, where effective damage identification can still be achieved for the remaining working conditions. Furthermore, SNRs of both methods remain above 10, indicating favorable signal detectability. Meanwhile, the localization errors are controlled within 7 mm.

To further quantify the relative performance between modal shape curvature and ULS curvature under identical working conditions, three auxiliary ratio indices are introduced. *R*_PBR_ denotes the ratio of the PBR calculated using modal shape curvature to the corresponding PBR obtained from ULS curvature. Similarly, *R*_Eloc_ and *R*_SNR_ denote the ratios of localization error *E*_loc_ and SNR calculated from modal shape curvature to their corresponding values obtained from ULS curvature.

Comparing the corresponding data in Table 2 and Table 3, the results are shown in Table 4. A value of *R*_Eloc_ lower than unity represents better localization accuracy of modal shape curvature, while values of *R*_PBR_ and *R*_SNR_ greater than unity indicate that modal shape curvature outperforms ULS curvature in damage saliency and anti-noise capability.

For the two methods in Table 2 and Table 3, the metric ratio of modal shape curvature to ULS curvature was calculated under the same damage configuration and delamination depth. Taking the surface smoothing algorithm as an example, it can be seen from Table 4 that the PBR of modal shape curvature is 1.29–1.47 times that of ULS curvature, its localization error accounts for only 0.78–0.83 of ULS curvature, and SNR reaches 1.23–1.52 times.

On the other hand, from Table 4, we can also see that compared with the surface smoothing algorithm, the curvature mode change rate method has a higher *R*_PBR_, indicating its slight superiority in modal curvature characterization and more prominent advantage in damage localization. The two methods achieve similar SNR improvement performance in terms of *R*_SNR_.

To further quantitatively compare the performance between the two baseline-free algorithms, the ratios of metrics (Table 3 of curvature mode change rate method/Table 2 of surface smoothing algorithm) under identical working conditions were calculated. The comparison results are shown in Table 5.

From Table 5, it can be seen that the PBR of the curvature mode change rate method is 1.51–1.99 times that of the surface smoothing algorithm, while its positioning error is reduced to 0.50–0.79 of the latter. The corresponding SNR has increased by 1.22 to 1.36 times. For two types of damage and three types of damage, this performance advantage remains consistent for boundary-layer and intermediate-layer delamination, as well as for modal shape curvature and ULS curvature indicators.

By analyzing the data in Table 2, Table 3, Table 4 and Table 5, combined with the damage index distributions illustrated in Figure 4, Figure 5, Figure 6 and Figure 7, the following conclusions can be drawn:

(1) For identical algorithm and curvature indicator, boundary-layer delamination (ply1–ply2) produces higher PBR and SNR, as well as smaller localization error than middle-layer delamination (ply4–ply5). This quantitatively confirms that near-surface delamination yields more detectable damage features than middle-thickness inter-ply damage.
(2) Under the same damage configuration and algorithm, modal shape curvature consistently achieves higher PBR, higher SNR and lower localization error compared with ULS curvature. Even though ULS curvature-based methods can identify delamination in numerical simulation, its relatively low PBR and SNR indicate that its damage features are vulnerable to background noise, which may lead to unreliable damage identification outcomes.
(3) Comparing the two baseline-free algorithms under the same curvature input, the curvature mode change rate method yields larger PBR and SNR and smaller localization error than the surface smoothing algorithm across all simulation cases, demonstrating superior delamination detection performance.

## 4. Experimental Validation

In this work, experiments are carried out to verify the feasibility of the proposed strategy for identifying delamination defects in composite laminates.

### 4.1. Experimental Specimen

The specimen was fabricated as an eight-layer conventional carbon-fiber composite laminate. Its geometric dimensions and ply orientations were consistent with those of the numerical simulation model. The plate dimensions depicted in Figure 8 were determined considering manufacturing and experimental constraints. A fixed-end boundary condition was implemented in the test, and a clamping section was therefore reserved at the left end of the specimen.

Laser measurement technology was adopted for experimental testing. The vibration velocity and displacement at measuring points can be derived by analyzing the frequency shift in laser signals reflected from the structural surface. Owing to diffuse reflection from bare carbon-fiber surfaces, the laser beam could not be properly focused and valid vibration signals could not be captured. To solve this problem, pretreatment was performed on the specimen prior to tests. Segmented reflective-film patches were pasted at individual measuring-point positions to improve surface reflectivity. Each piece of reflective film was cut into small sizes merely covering one measurement point instead of covering the whole plate, aiming to limit additional added mass. The total extra mass introduced by these discrete small patches is very limited. As shown in Table 6, the relative errors between experimental and simulated natural frequencies are less than 2%, which indicates that the global dynamic and modal characteristics of the laminate are barely disturbed by the reflective-film patches. Measuring points were marked at preset positions. The treated specimen is shown in Figure 9.

To simulate realistic damage scenarios, two damage cases were designed: (a) single delamination, and (b) double delamination. The dimensions and locations of these delaminations are illustrated in Figure 10.

Artificial delamination defects were prefabricated by embedding polytetrafluoroethylene (PTFE, Teflon) thin-film inserts during the hand lay-up manufacturing process. The PTFE insert has a thickness of 0.02 mm. For the specimen with one delamination defect, the insert dimension is 100 mm × 60 mm. For the specimen containing two delamination defects, each insert measures 30 mm × 30 mm. All PTFE films were embedded between the first and second ply (near-surface boundary-layer interface).

It must be clarified that the layered arrangement in numerical simulation is designed for parameter comparison of damage identification performance. The damage layout of the test specimens has been adjusted to adapt to the actual processing limitations, reserve clamping areas, and the scanning measurement point grid requirements of the Laser Doppler Vibrometer. Therefore, the experimental verification in this article is not an accurate one-by-one reproduction of simulation examples. The main purpose is to verify the overall feasibility of the proposed baseline-free curvature class algorithm in identifying near-boundary-layer delamination damage under actual measurement noise interference.

### 4.2. Test System and Process

During the experimental testing process, the specimen was excited by a vibration exciter and one edge of the plate was clamped. As validation experiments, two kinds of damage types as shown in Figure 10 were selected. The measurement system is shown schematically in Figure 11a, and the actual test system is shown in Figure 11b.

The scanning Laser Doppler Vibrometer (PSV-400, Polytec GmbH, Waldbronn, Germany) was adopted for modal parameter acquisition. An exciter was utilized to apply random excitation to the cantilevered composite laminate. The excitation signal was generated by the control system of the Laser Doppler Vibrometer. A grid of 13 × 14 (182 total) measurement points was predefined on the specimen surface for laser scanning. During the vibration test, the laser was controlled to scan each measurement point and feed-back the vibration information. Key acquisition parameters were set as: measurement frequency range 0–2000 Hz, sampling frequency 5120 Hz, FFT frequency resolution 1 Hz, and 25-time time-domain averaging for noise reduction. The control and built-in modal analysis module of PSV-400 was used to compute modal frequencies and mass-normalized modal shape data.

### 4.3. Measurement Signal

Vibration measurement of laminated plate specimens without damage is carried out. The first several modes of laminated plate specimens are extracted from the vibration response parameters processed by the modal analysis system. The results are compared with the numerical simulation results under the same conditions. The results are shown in Figure 12 and Figure 13.

Analysis of Figure 12 and Figure 13 shows that under the same material, structure and loading mode, the measured frequencies and modal shapes of the first several modes are not significantly different from the numerical simulation results.

To further quantitatively evaluate the agreement between numerical simulation and experimental test results, the relative errors of natural frequencies between simulated and measured data are calculated in this study. For modal shape comparison, the Modal Assurance Criterion (MAC) is adopted to achieve quantitative assessment of modal shape correlation.

The relative percentage error for natural frequency is expressed as:

(11) $$\varepsilon_{f}=\frac{\left|f_{\mathrm{num}}-f_{\exp}\right|}{f_{\exp}}\times 100\%$$

where $f_{\mathrm{num}}$ and $f_{\exp}$ are natural frequencies obtained from numerical simulation and experimental test, respectively.

The Modal Assurance Criterion quantifies the correlation between simulated modal shape vector $\phi_{s}$ and experimental modal shape vector $\phi_{e}$:

(12) $$\mathrm{MAC}\left(s,e\right)=\frac{{\left|\phi_{s}^{T}\phi_{e}\right|}^{2}}{\left(\phi_{s}^{T}\phi_{s}\right)\left(\phi_{e}^{T}\phi_{e}\right)}$$

MAC values range from 0 to 1; a value approaching 1 represents high consistency between two modal shapes.

The first-four-order natural frequencies and modal shapes obtained from numerical simulation and experimental measurements are substituted into Equations (11) and (12), and the corresponding calculation results are listed in Table 6.

**Table 6:** Relative frequency errors and MAC values for the first four modes of the intact laminate.

Mode No.	$f_{\exp}$/Hz	$f_{\mathrm{num}}$/Hz	$\varepsilon_{f}$/%	MAC
1	81.24	82.12	1.08	0.942
2	107.87	109.23	1.26	0.924
3	200.86	203.39	1.26	0.921
4	301.07	305.94	1.62	0.897

Quantitative comparison in Table 6, together with visual inspection from Figure 12 and Figure 13, demonstrates reasonable agreement between numerical and experimental modal results. The relative frequency errors for the first four modes are all below 2%, and most MAC coefficients exceed 0.90.

For various damaged specimens, the specimen dimensions, material properties, boundary constraints and loading conditions are consistent with those of intact specimens, and only minor discrepancies can be observed between simulation and experimental results. To avoid presenting excessive similar comparison results, only the comparison results of intact specimens were demonstrated herein, which verifies the reliability of the simulation model and experimental data and provides support for the subsequent structural damage identification analysis.

### 4.4. Damage Recognition Results Based on Measured Signals

The measured frequency and modal shape data under two damage conditions were extracted to calculate the modal shape curvature and ULS curvature. Subsequently, the damage identification indices based on the surface smoothing algorithm and curvature mode change rate method were calculated according to Equations (4) and (7) respectively. As demonstrated by the simulation results in the previous section, although the ULS curvature-based method exhibits satisfactory delamination identification capability in numerical simulations, its low amplitude damage indices are highly sensitive to measurement noise. Reliable experimental validation of the ULS curvature-based method cannot be achieved under the present experimental conditions. For this reason, in the subsequent calculations, this article only uses the modal shape curvature to process the measured experimental data. The corresponding damage maps are shown in Figure 14 and Figure 15.

The above three quantitative metrics PBR, *E*_loc_ and SNR are further calculated for experimental measurement data. Only modal shape curvature is processed for experimental specimens, since ULS curvature-based indices are severely corrupted by measurement noise and cannot deliver valid damage location results in the present test. Quantitative experimental results are listed in Table 7; the comparison results of the two methods are shown in Table 8.

As shown in Table 7 and Table 8 and Figure 14 and Figure 15, experimental results are consistent with the numerical conclusions. Owing to measurement noise and experimental uncertainties, the experimental PBR and SNR decrease and localization error increases relative to numerical simulation. Nevertheless, the curvature mode change rate method still maintains higher PBR-SNR values and lower localization error than the surface smoothing algorithm for real test specimens.

## 5. Conclusions

In this work, two existing curvature-based parameters, i.e., modal shape curvature and ULS curvature, are adopted to investigate delamination damage in eight-ply symmetric cross-ply composite laminates. Two baseline-free damage identification approaches, namely the surface smoothing algorithm and the curvature mode change rate method, are implemented and comparatively evaluated using both a damage index mapping approach for visual inspection and three quantitative evaluation metrics—PBR, localization error *E*_loc_, and SNR—for objective performance assessment. Distinct from previous studies that applied these curvature-related techniques separately, the present work systematically compares the two curvature indicators alongside the two identification algorithms, explores the effect of delamination depth (i.e., boundary-layer and middle-layer delamination), and further carries out experimental validation on carbon-fiber composite laminate specimens under measurement noise interference. The main conclusions are drawn as follows:

(1) Based on curvature-related damage indicators, both the surface smoothing algorithm and curvature mode change rate method can effectively identify delamination damage without intact structure baseline data. Quantitative evaluation via PBR, *E*_loc_ and SNR demonstrates that boundary-layer delamination generates more prominent damage features with higher PBR and SNR and smaller localization error than middle-layer delamination. For boundary-layer delamination (ply1–2), the curvature mode change rate method achieves a maximum PBR of 32.12, SNR of 35.49 dB, and a minimal *E*_loc_ down to 1.54 mm; for middle-layer delamination (ply4–5), the corresponding maximum PBR decreases to 28.52, SNR drops to 33.87 dB, and the minimal *E*_loc_ increases to 1.74 mm.
  This depth-related finding is supported solely by numerical simulation and has not been verified by experiments, since no physical specimens with middle-layer delamination were prepared in the present tests. The experimental verification in this study is limited to the case of near-surface boundary-layer stratification. The experimental evaluation of the depth effect of delamination will be the focus of our future research.
(2) Modal shape curvature-based approaches outperform ULS curvature-based schemes in terms of PBR, SNR and localization accuracy. Under identical simulation conditions, modal shape curvature yields 1.29–1.53-fold higher PBR and 1.23–1.52-fold higher SNR compared with ULS curvature counterparts, accompanied by reduced localization error. Although ULS curvature performs well in noise-free numerical simulation, its low PBR and damage indictors make damage features easily submerged by practical measurement noise; consequently, reliable experimental identification and validation cannot be realized under the present test conditions.
(3) Among the two investigated baseline-free algorithms for composite laminate delamination identification, the curvature mode change rate method exhibits higher damage sensitivity than the surface smoothing algorithm. In numerical simulations (Table 5), it yields 1.51–1.99 times higher PBR, 0.50–0.79 times lower localization error, and 1.22–1.36 times higher SNR. Consistent superiority is also observed in experimental analysis (Table 8), where it achieves 1.15–1.21 times higher PBR, 0.75–0.80 times lower localization error, and 1.14–1.24 times higher SNR relative to the surface smoothing algorithm. Such performance advantages hold for boundary-layer and middle-layer delamination, two damage types, and three damage cases, as well as modal shape curvature and ULS curvature indicators.
(4) The modal correlation between experimental testing and simulation shows good consistency. The relative frequency errors for the first four modes are all below 2%, and most MAC coefficients exceed 0.90. Owing to measurement noise and experimental uncertainties, the damage recognition performance based on experimental data has decreased compared to numerical simulations; nevertheless, modal shape curvature-based baseline-free approaches can still locate delamination approximately. Future work will focus on experimental investigation of middle-layer delamination.

## Figures and Tables

**Figure 1:**
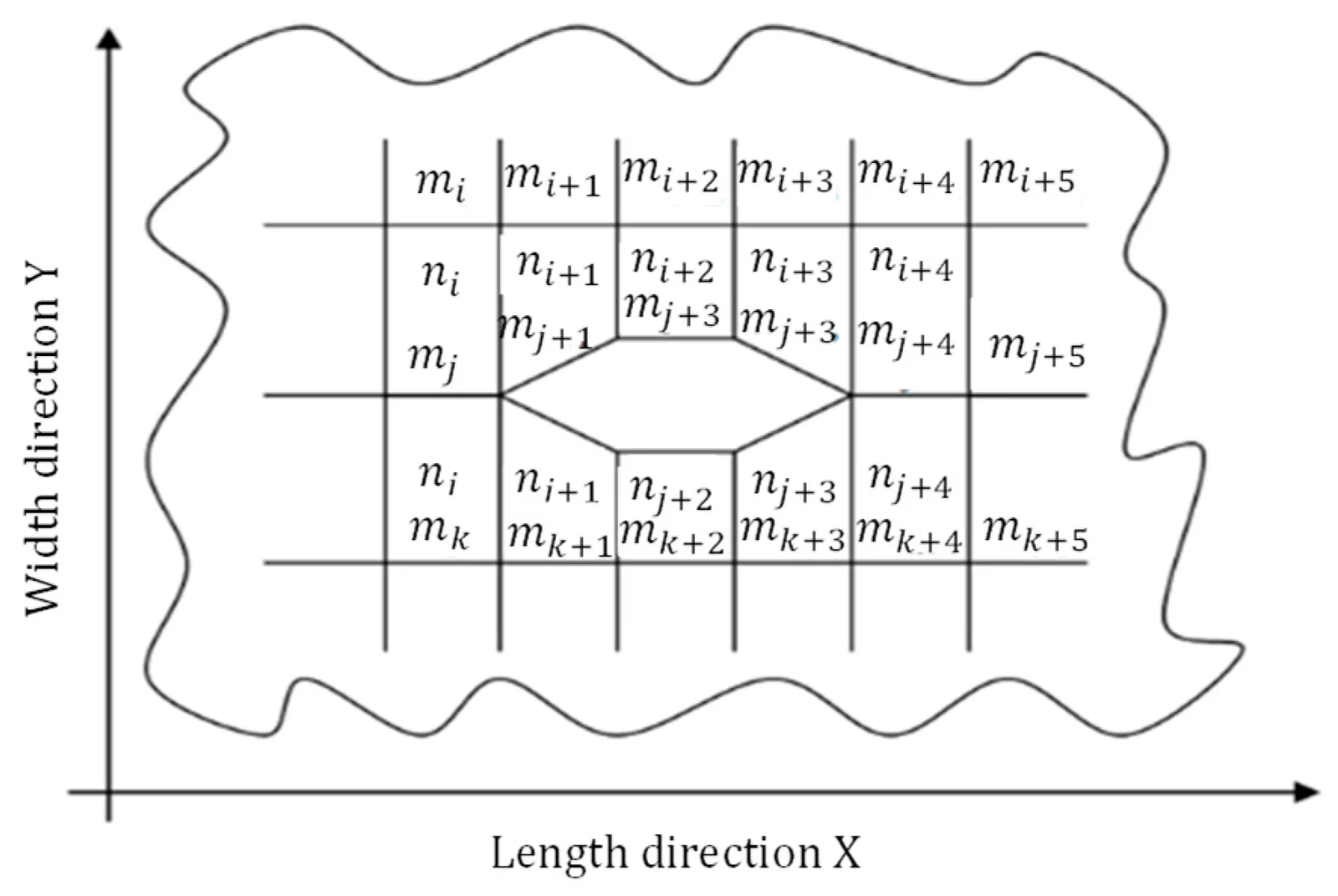
Node-merging method.

**Figure 2:**
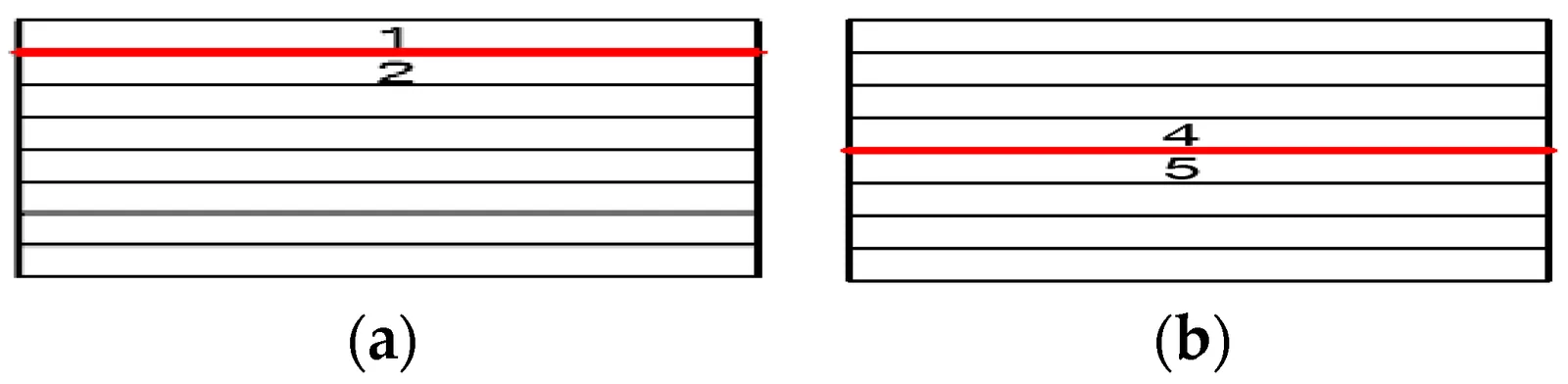
Schematic diagram of delamination damage in different layers. (**a**) Delamination damage between the first and second plies. (**b**) Delamination damage between the fourth and fifth plies.

**Figure 3:**
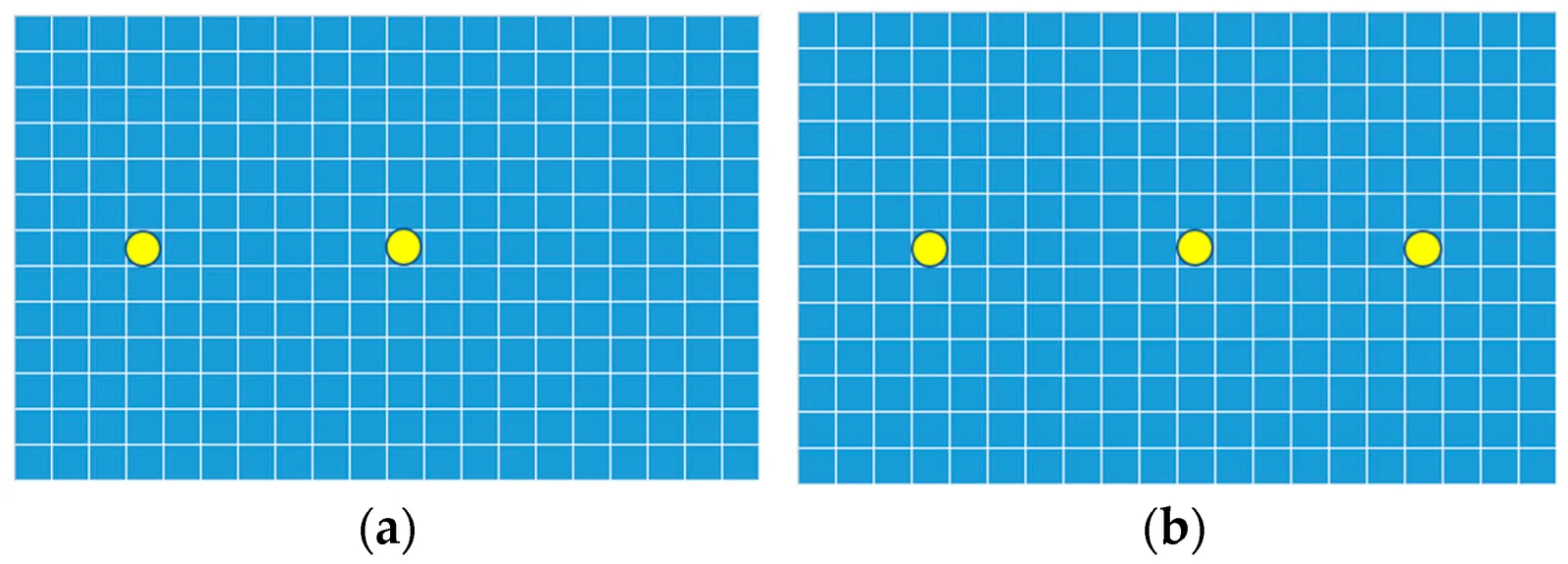
Numerical models for feasibility analysis. (**a**) Structure with two damages. (**b**) Structure with three damages.

**Figure 4:**
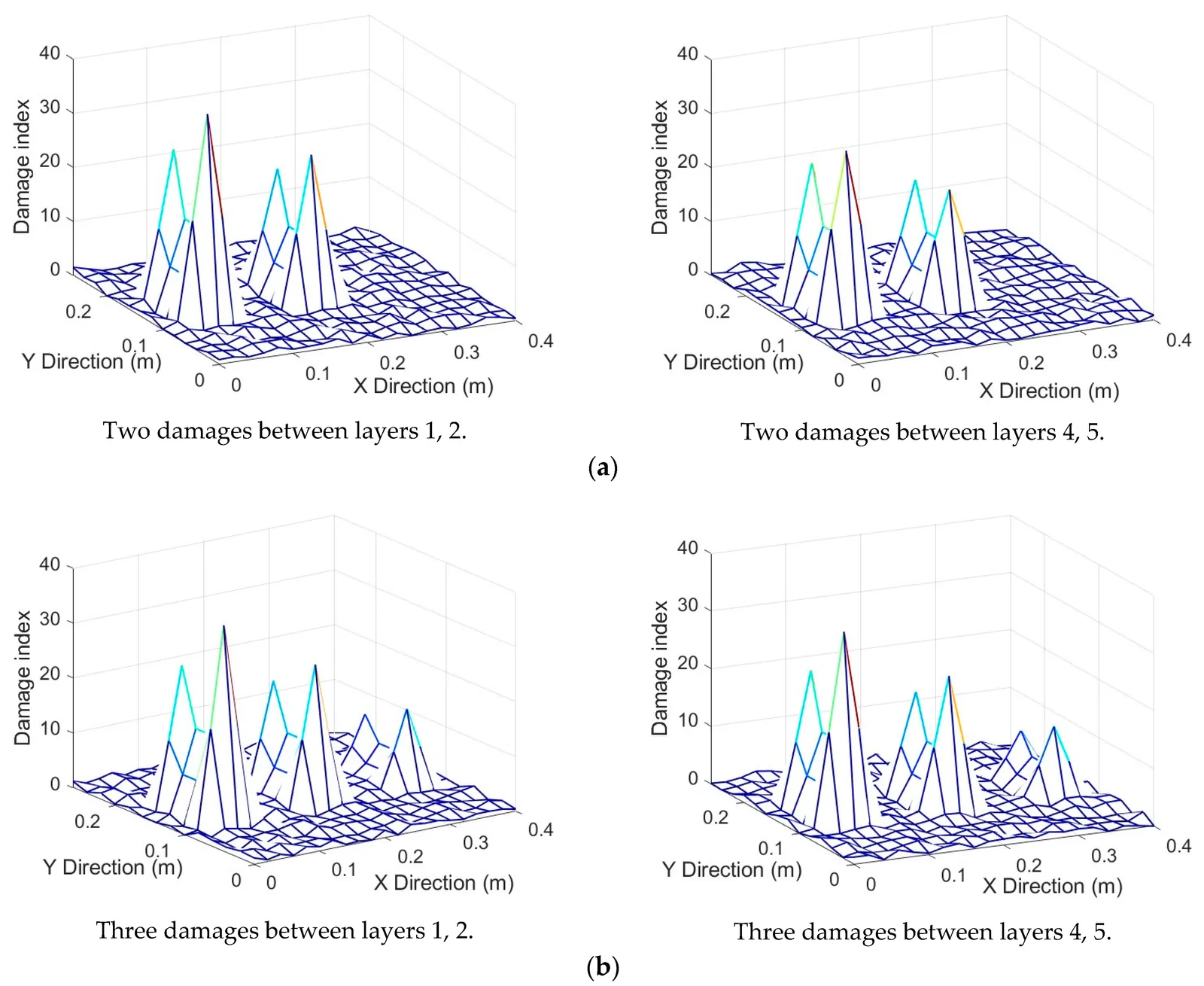
Damage recognition of laminated plates based on modal shape curvature and surface smoothing algorithms. (**a**) Two-damages recognition results. (**b**) Three-damages recognition results.

**Figure 5:**
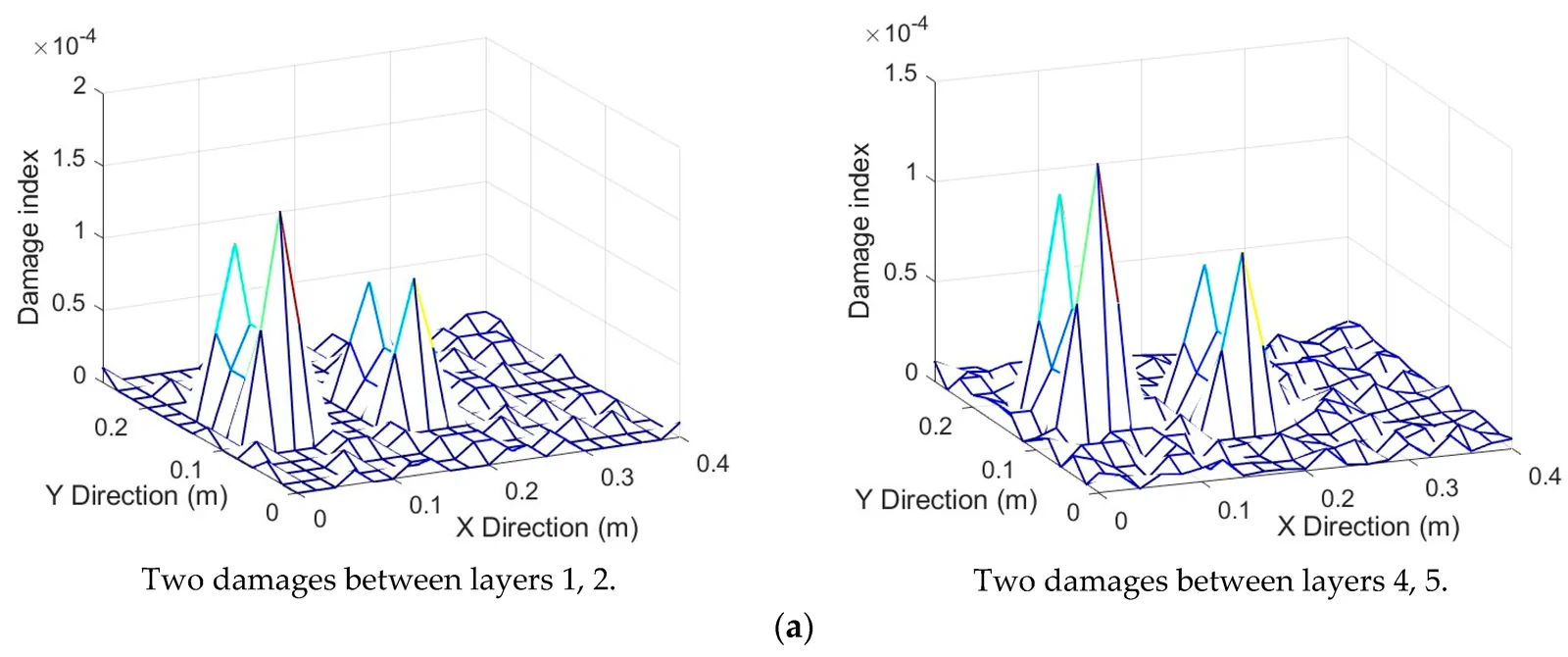

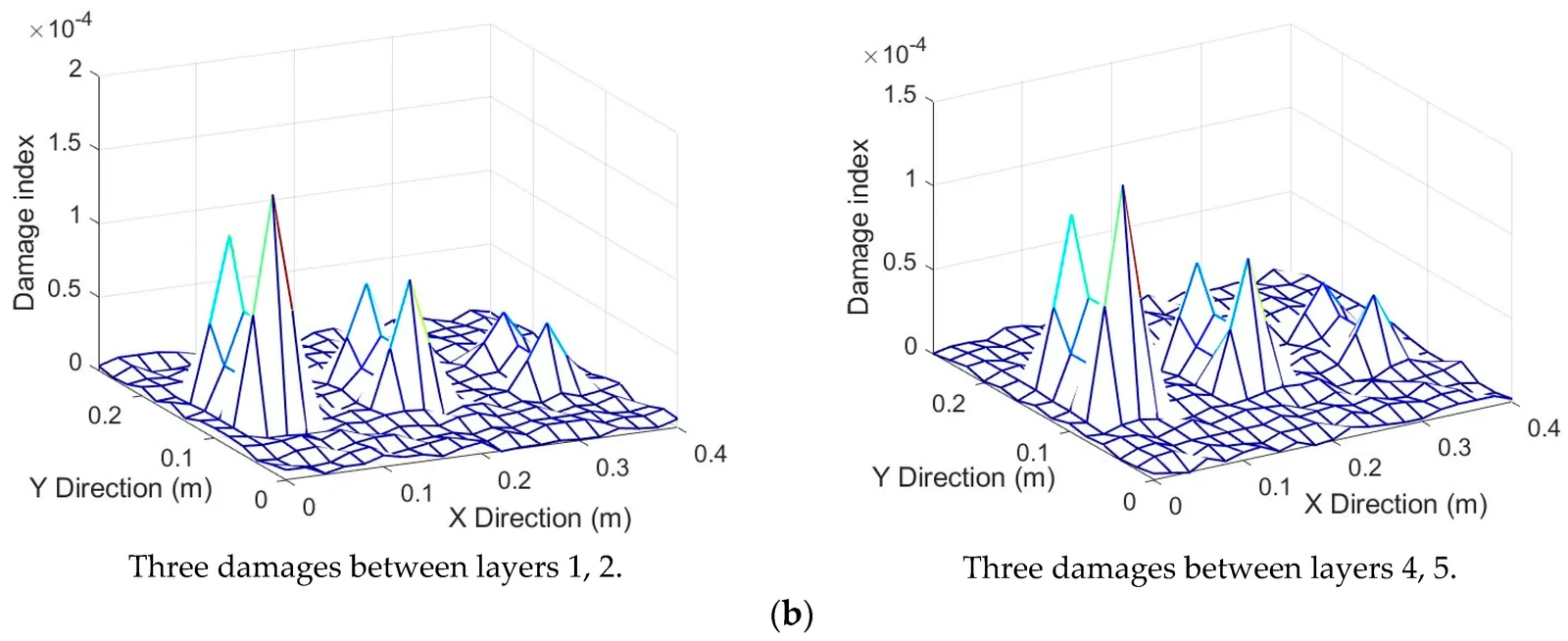
Damage recognition of laminated plates based on ULS curvature and surface smoothing algorithms. (**a**) Two-damages recognition results. (**b**) Three-damages recognition results.

**Figure 6:**
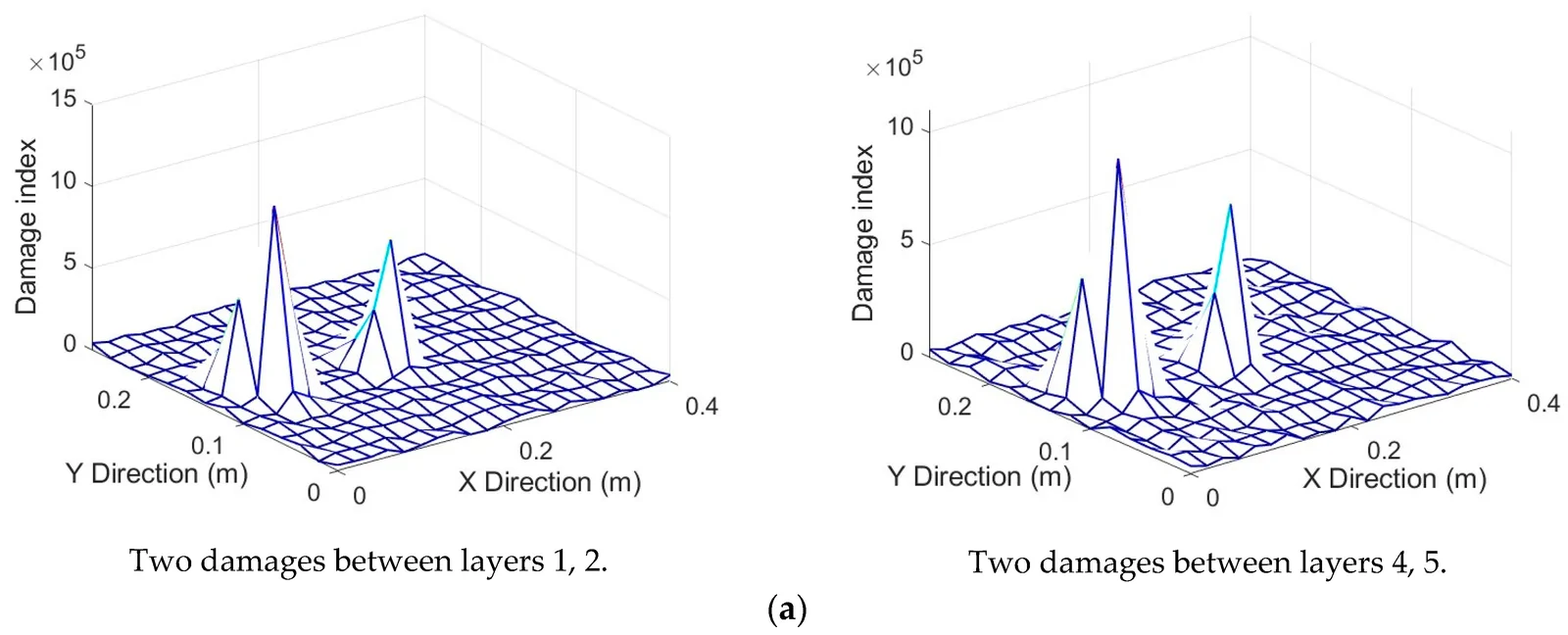

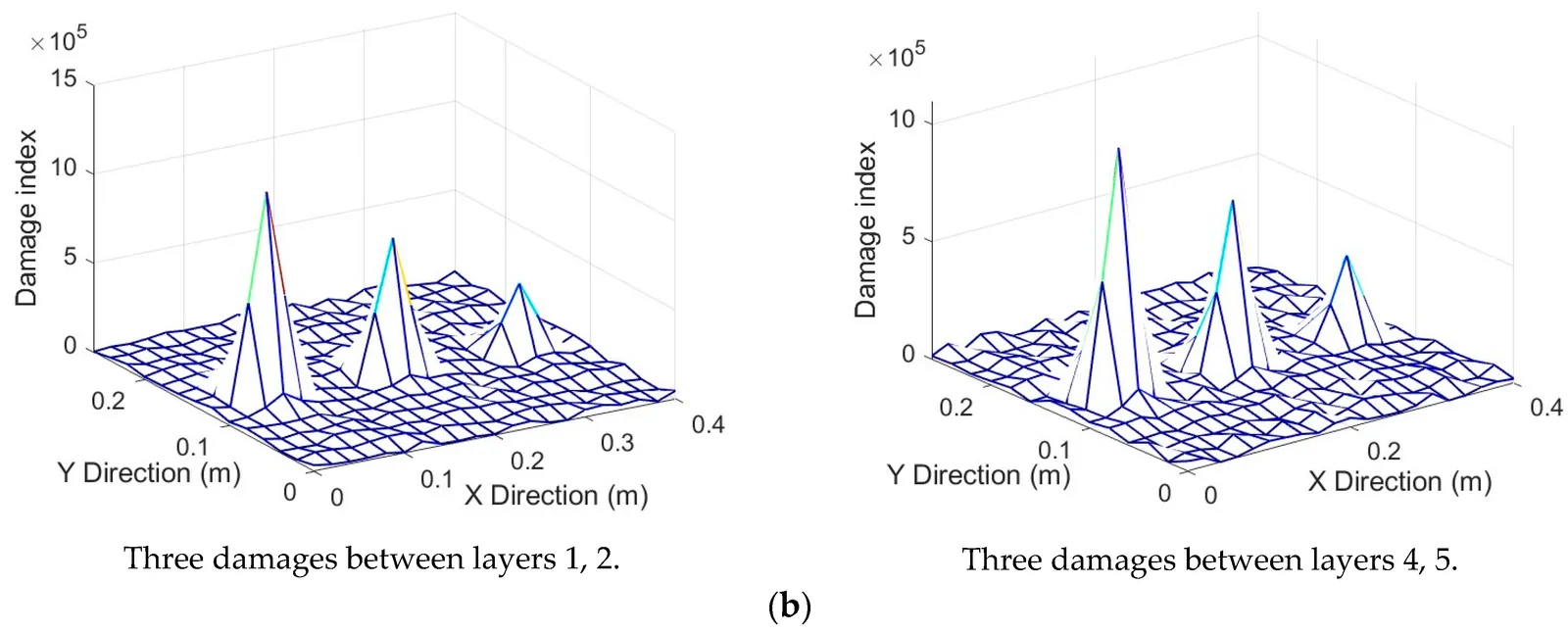
Damage recognition of laminated plates based on modal curvature change rate method. (**a**) Two-damages recognition results. (**b**) Three-damages recognition results.

**Figure 7:**
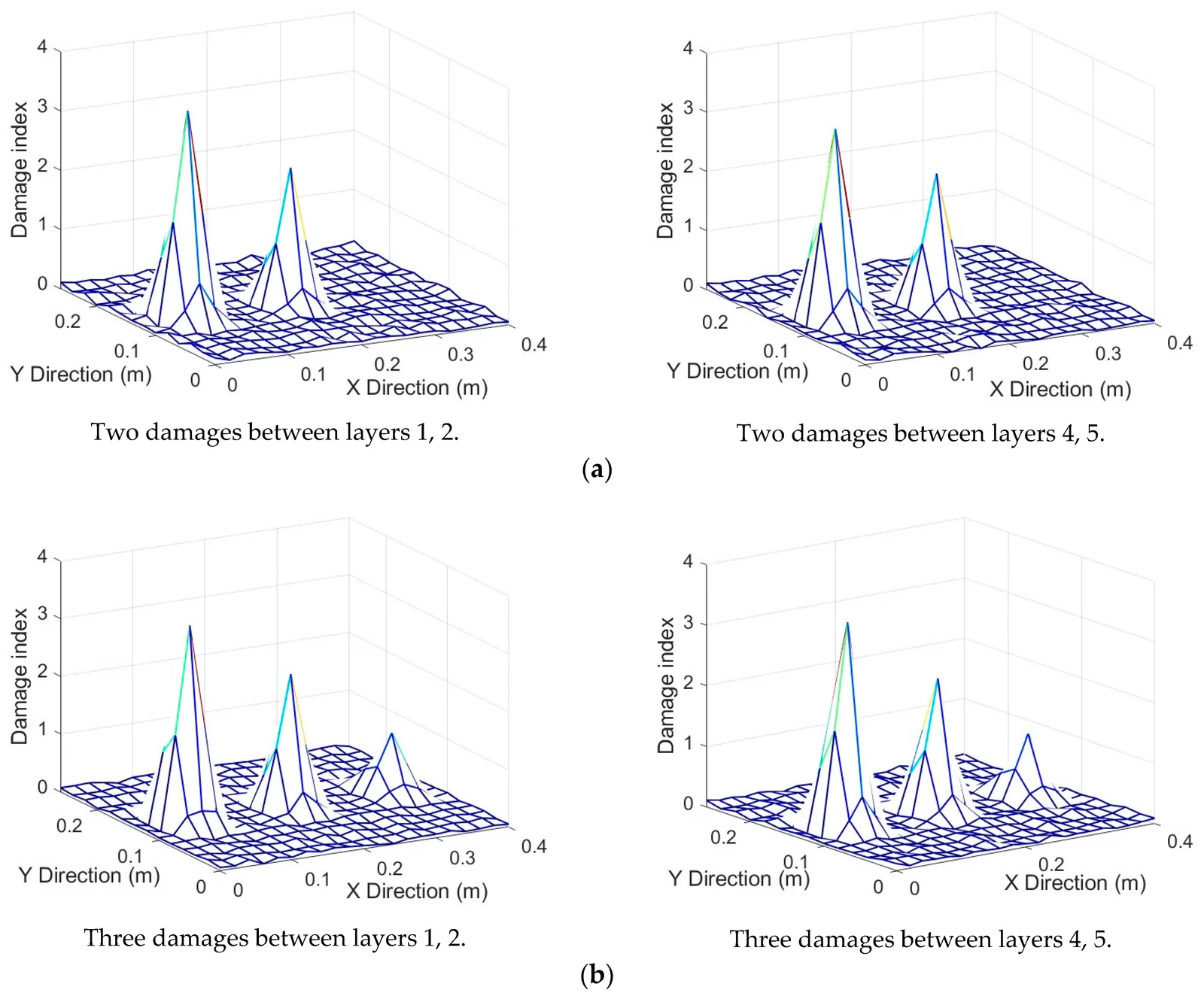
Damage recognition of laminated plates based on ULS curvature change rate method. (**a**) Two-damages recognition results; (**b**) three-damages recognition results.

**Figure 8:**
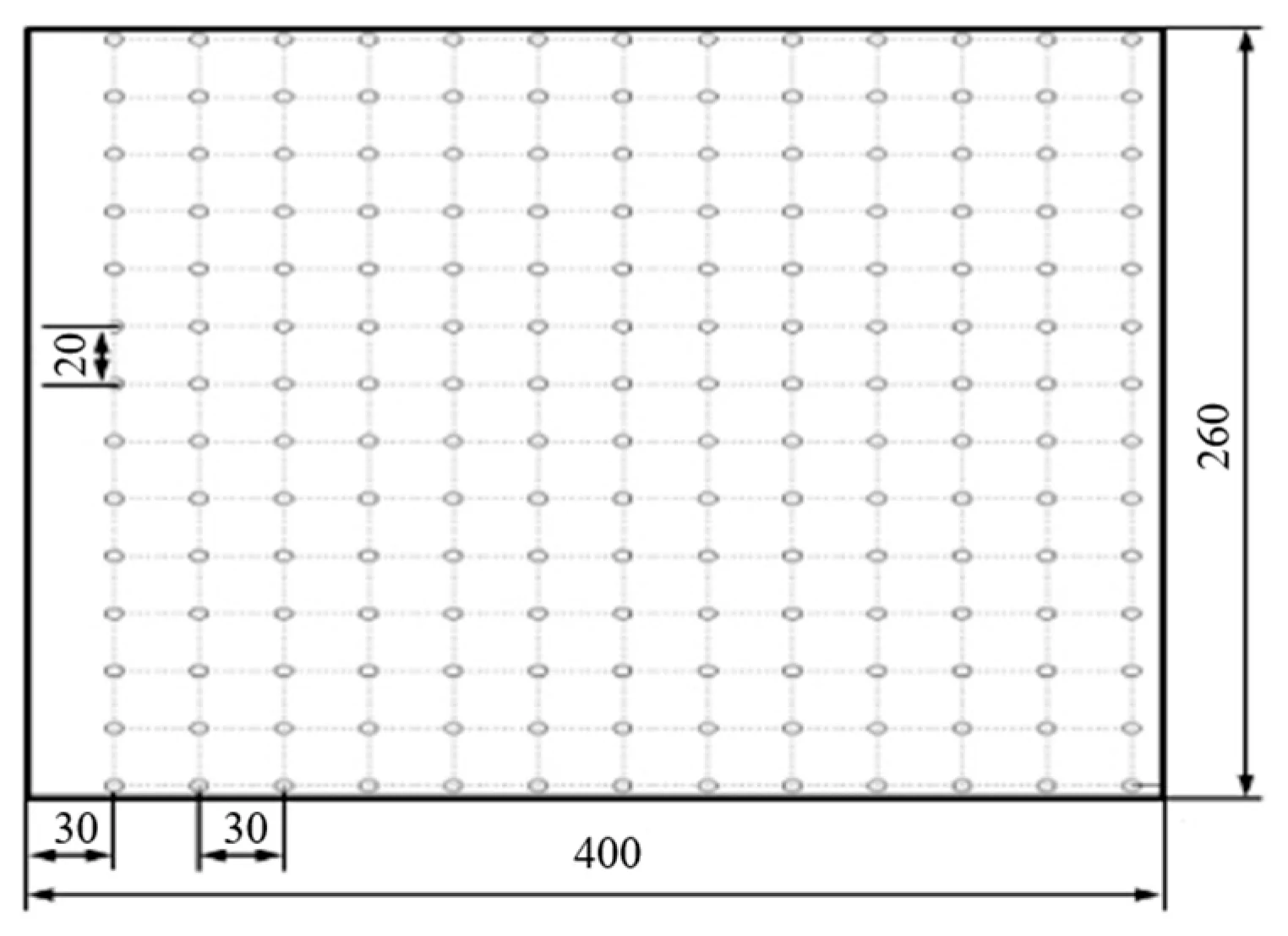
Design dimensions of laminates (unit: mm, plate thickness: 1.6 mm).

**Figure 9:**
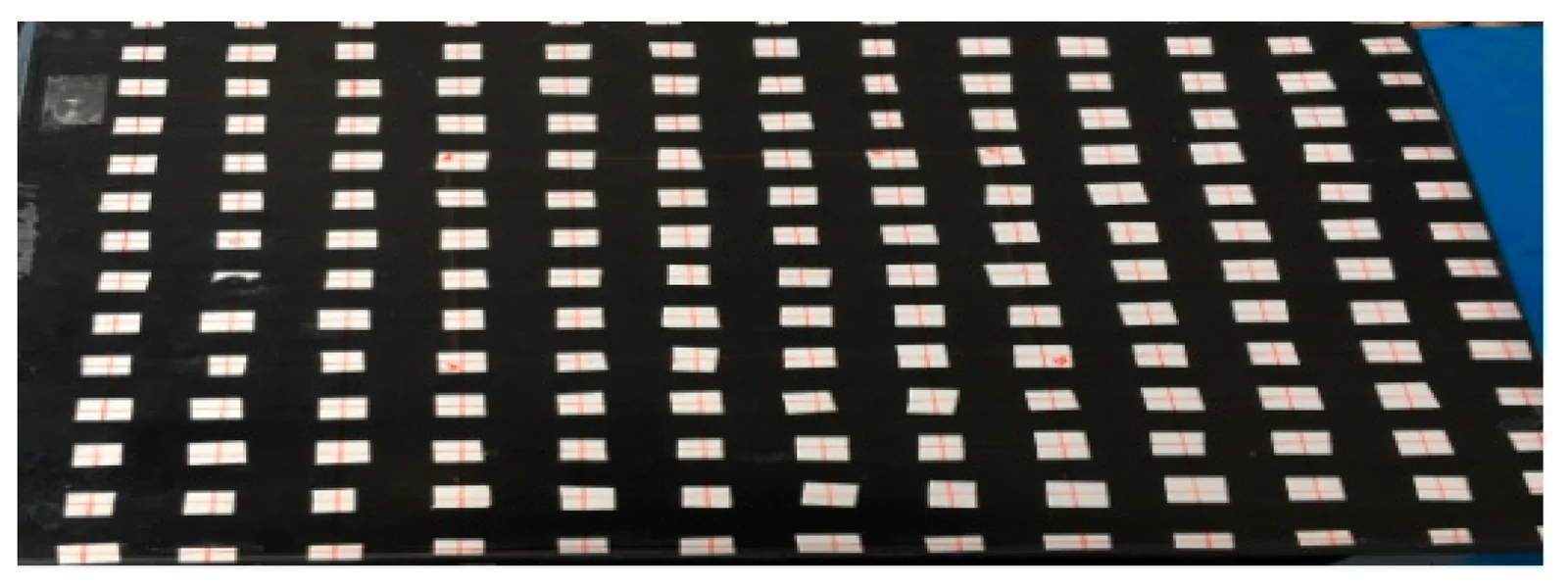
Pre-treated specimens.

**Figure 10:**
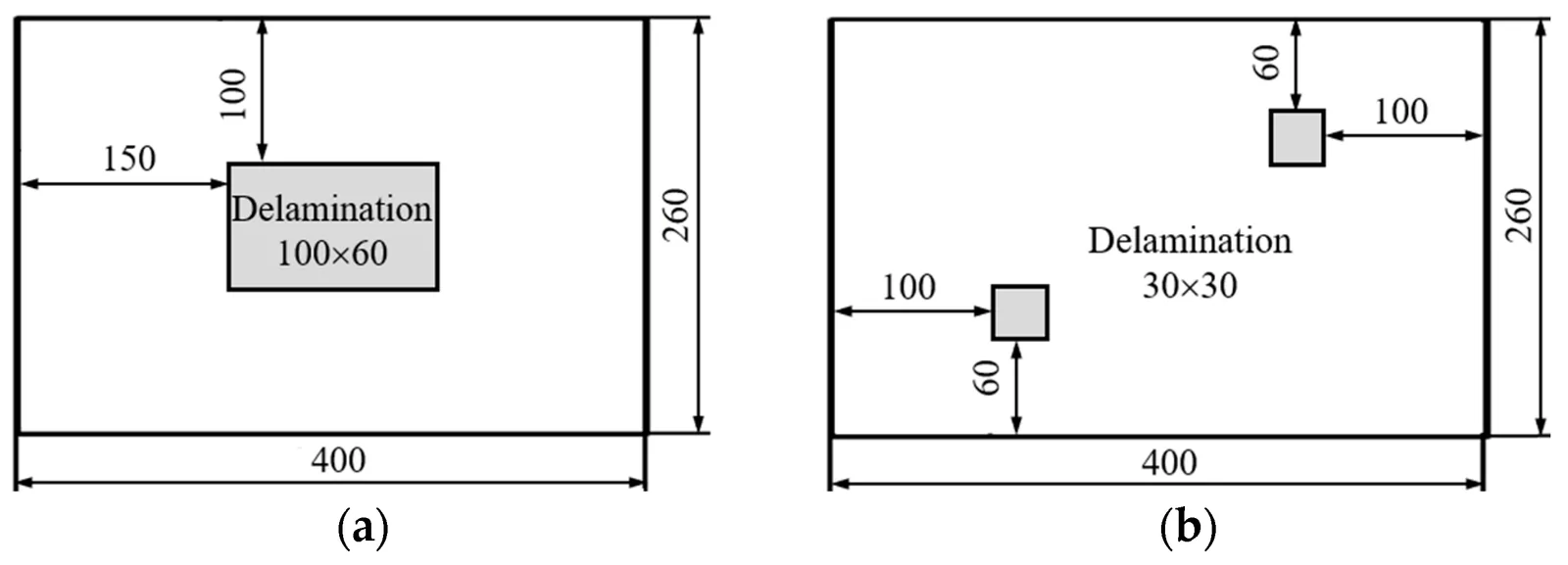
Presupposed damage location and number of laminated plates (unit: mm). (**a**) Structure with one delamination. (**b**) Structure with two delaminations.

**Figure 11:**
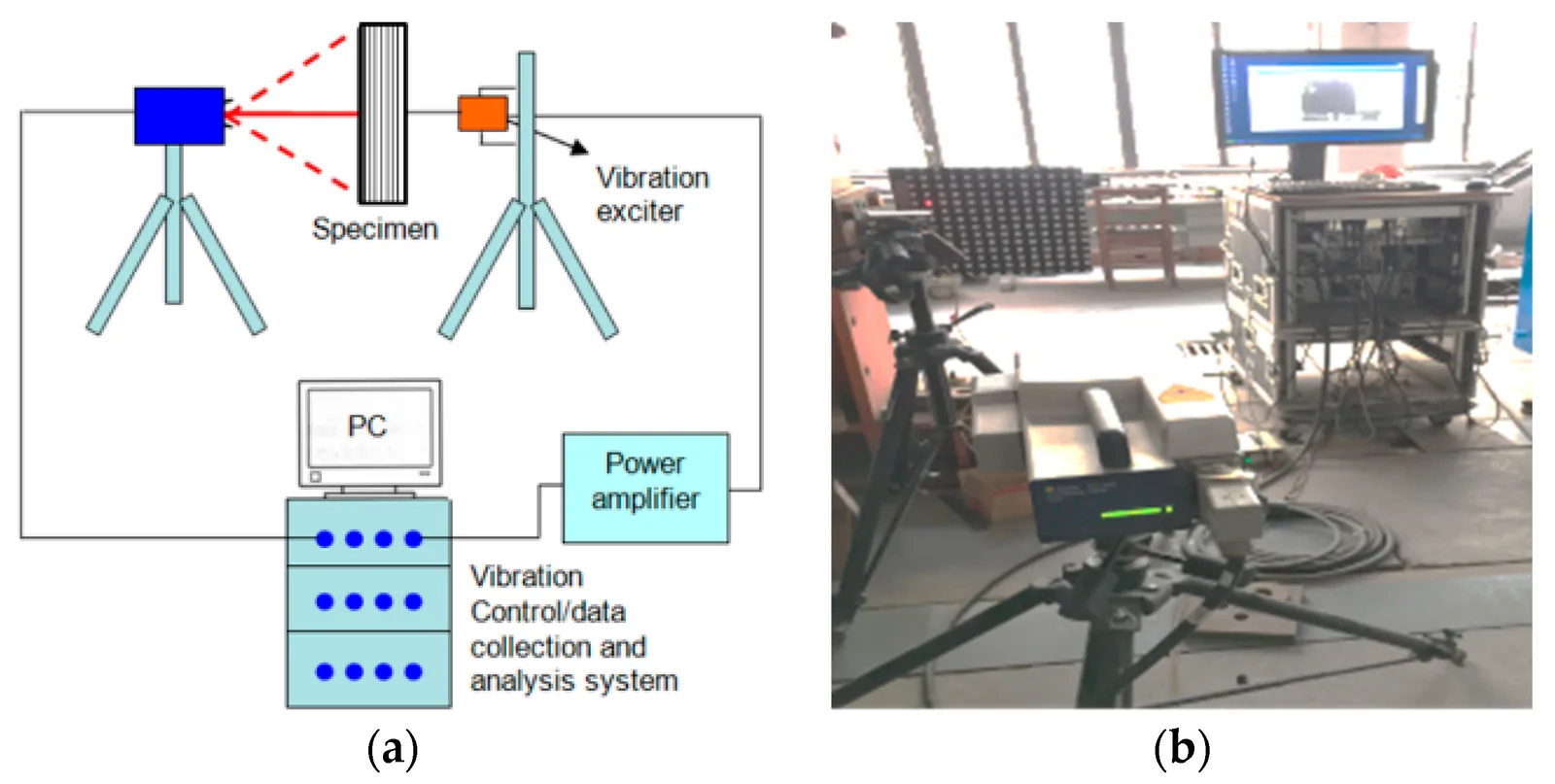
Vibration measurement system. (**a**) Sketch map of the vibration measurement system. (**b**) Practical experimental system.

**Figure 12:**
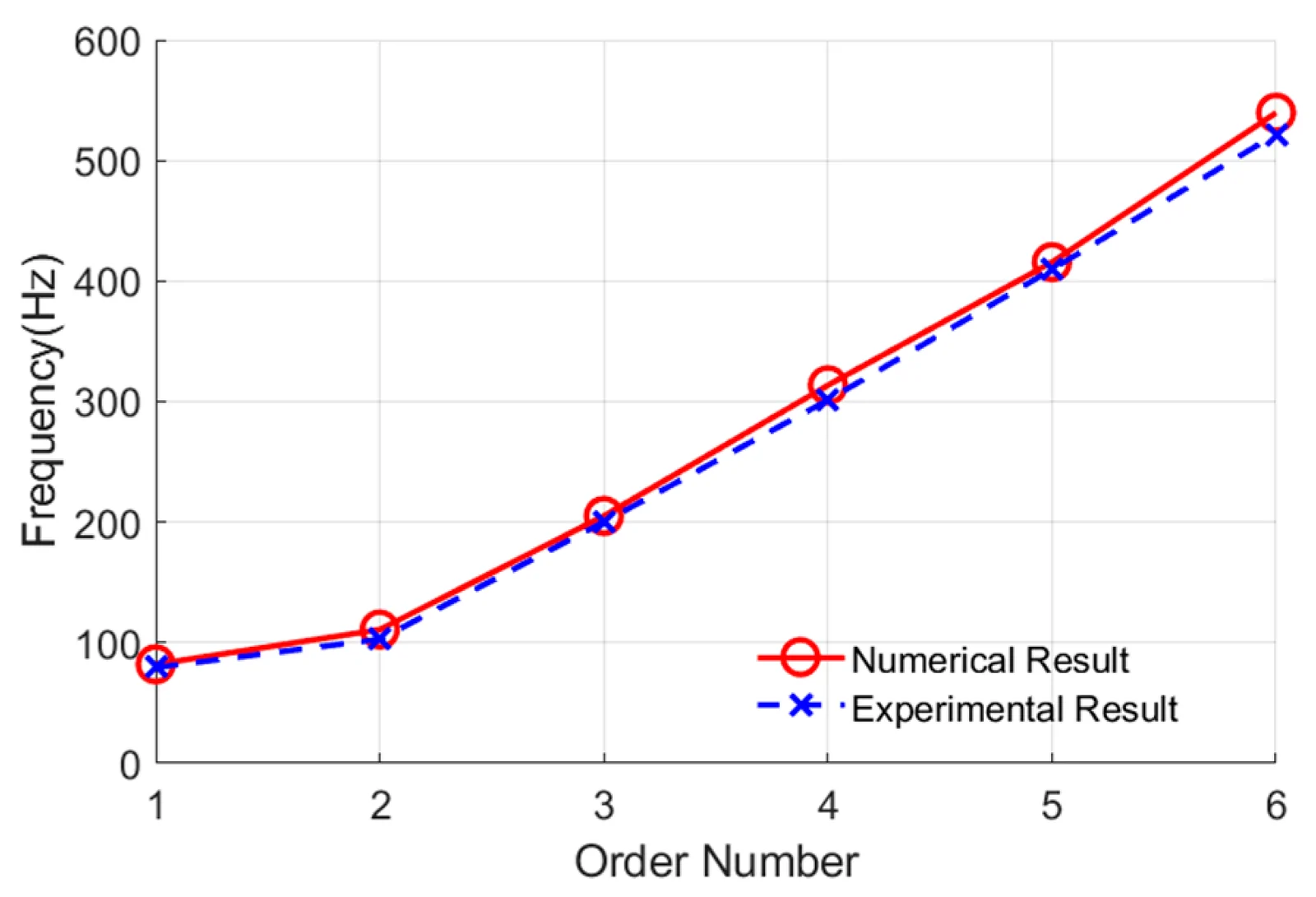
Comparisons of the first-six-order experimental and numerical frequencies of nondestructive laminates.

**Figure 13:**
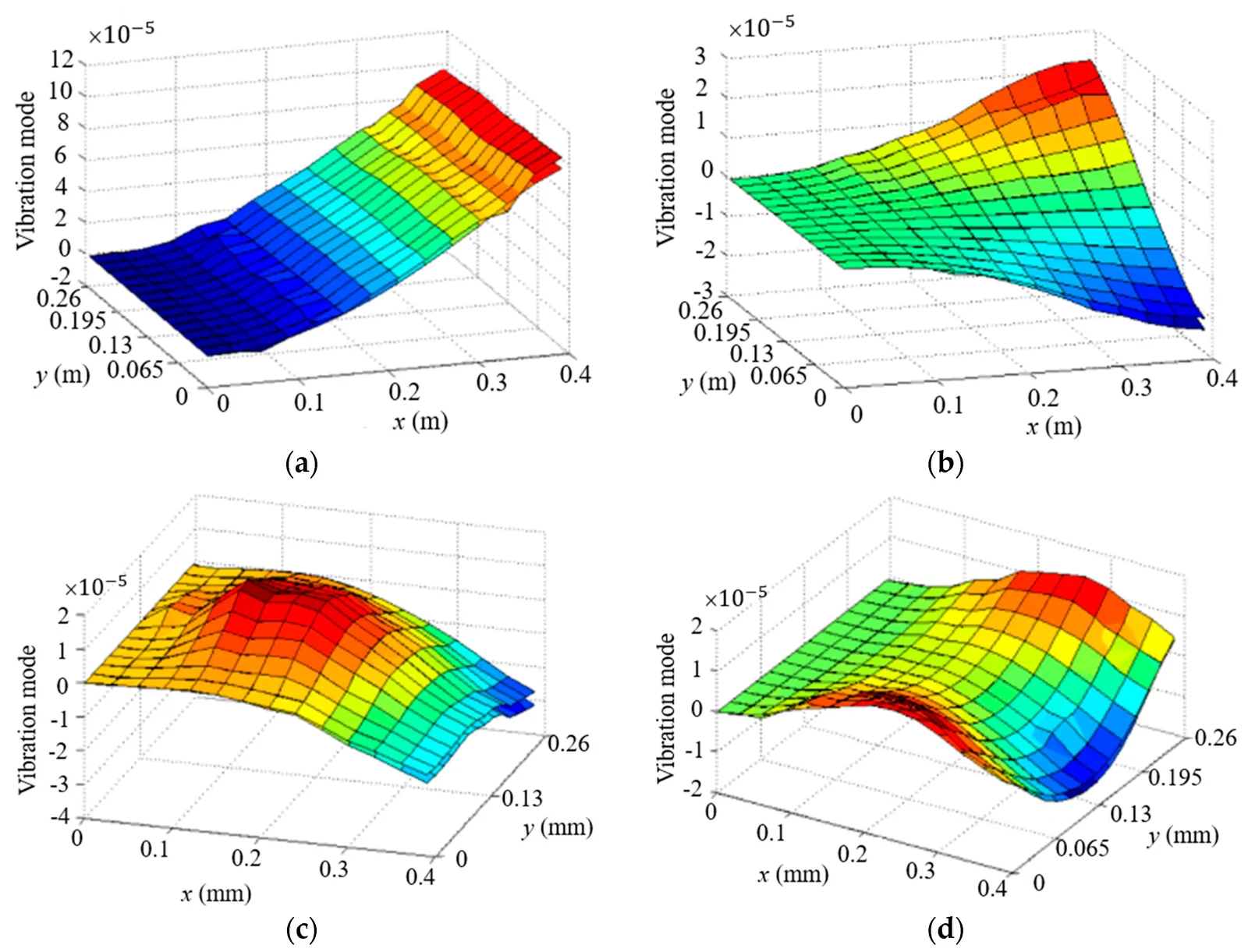
Comparisons between measured and simulated modes of the first four orders of nondestructive laminates. (**a**) First-order modal shapes. (**b**) Second-order modal shapes. (**c**) Third-order modal shapes. (**d**) Fourth-order modal shapes.

**Figure 14:**
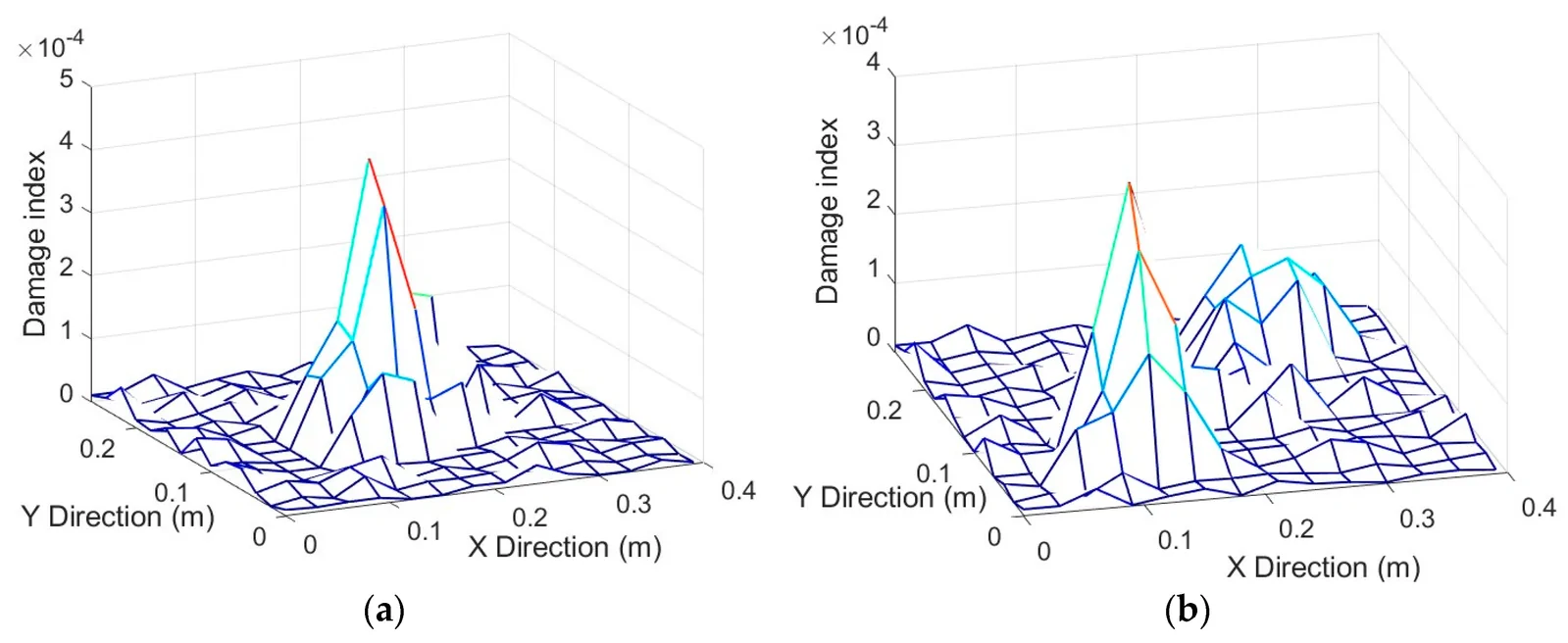
Damage recognition of laminated plates based on modal shape curvature and surface smoothing algorithms. (**a**) Structure with one delamination; (**b**) structure with two delaminations.

**Figure 15:**
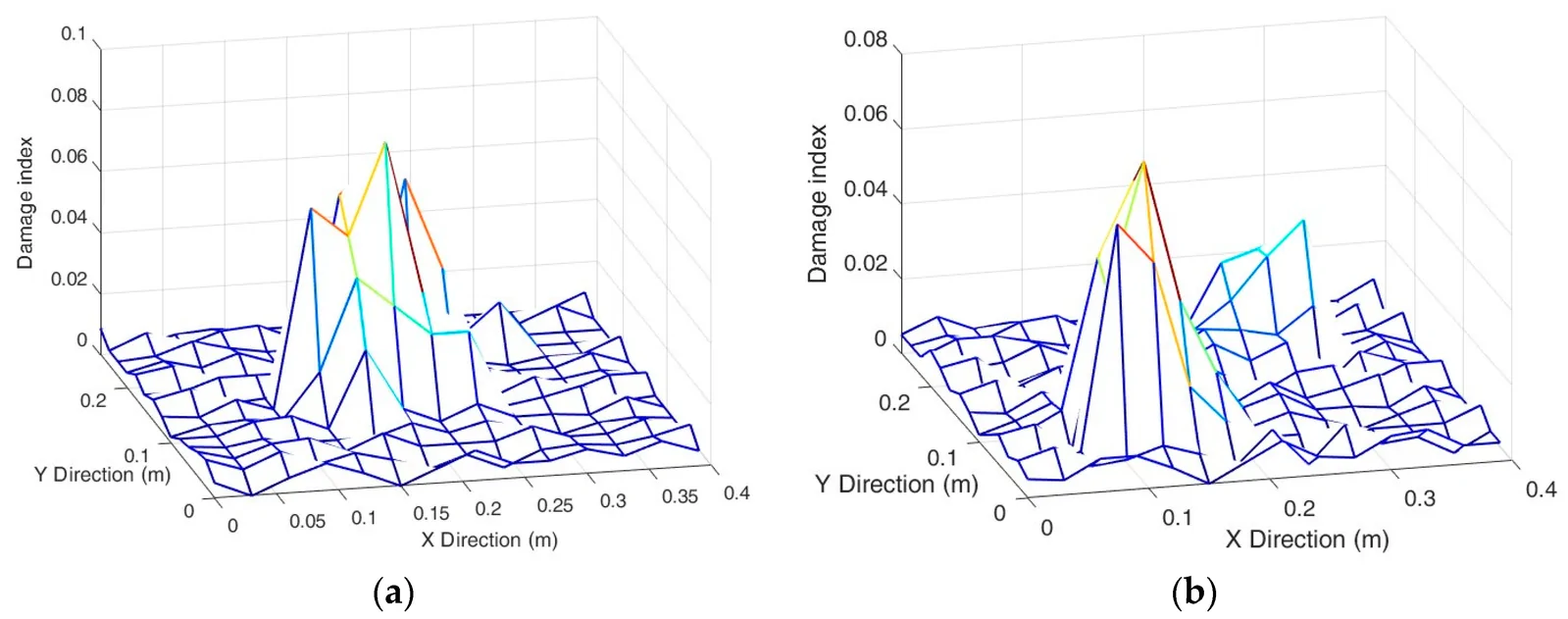
Damage recognition of laminated plates based on modal curvature change rate method. (**a**) Structure with one delamination; (**b**) structure with two delaminations.

**Table 1:** The first six modal frequencies of damaged and undamaged structures.

Order Number	Structure Without Damage/Hz	Structure with Damage/Hz	
		Between Layers 1, 2	Between Layers 4, 5
1	82.12	81.79	81.30
2	110.92	109.58	108.03
3	205.19	203.91	202.42
4	313.57	311.08	309.49
5	415.69	413.62	411.38
6	539.61	537.38	533.93

**Table 2:** Quantitative metrics for surface smoothing algorithm under different simulated delamination cases.

Case	Curvature Indicator	Delamination Location	PBR	Eloc/mm	SNR/dB
Two damages	Modal shape curvature	Boundary layer (ply1–ply2)	16.15	2.98	27.13
Two damages	Modal shape curvature	Middle layer (ply4–ply5)	14.51	3.46	24.92
Two damages	ULS curvature	Boundary layer (ply1–ply2)	11.39	3.62	21.07
Two damages	ULS curvature	Middle layer (ply4–ply5)	9.87	4.16	18.96
Three damages	Modal shape curvature	Boundary layer (ply1–ply2)	9.49	3.97	22.87
Three damages	Modal shape curvature	Middle layer (ply4–ply5)	8.47	5.32	21.79
Three damages	ULS curvature	Boundary layer (ply1–ply2)	7.08	5.11	18.55
Three damages	ULS curvature	Middle layer (ply4–ply5)	6.64	6.43	14.37

**Table 3:** Quantitative metrics for curvature mode change rate method under different simulated delamination cases.

Case	Curvature Indicator	Delamination Location	PBR	Eloc/mm	SNR/dB
Two damages	Modal shape curvature	Boundary layer (ply1–ply2)	32.12	1.54	35.49
Two damages	Modal shape curvature	Middle layer (ply4–ply5)	28.52	1.74	33.87
Two damages	ULS curvature	Boundary layer (ply1–ply2)	21.25	2.18	25.75
Two damages	ULS curvature	Middle layer (ply4–ply5)	18.79	2.81	23.57
Three damages	Modal shape curvature	Boundary layer (ply1–ply2)	16.62	2.47	28.93
Three damages	Modal shape curvature	Middle layer (ply4–ply5)	15.26	3.58	28.24
Three damages	ULS curvature	Boundary layer (ply1–ply2)	10.88	3.48	22.76
Three damages	ULS curvature	Middle layer (ply4–ply5)	10.04	5.09	19.56

**Table 4:** Calculation results of the ratio of three quantitative indicators based on modal shape curvature and ULS curvature (M1 and M2 respectively represent surface smoothing method and curvature mode change rate method).

Case	Delamination Location	***R***_PBR_		***R***_Eloc_		***R***_SNR_	
		M1	M2	M1	M2	M1	M2
Two damages	Boundary layer (ply1–ply2)	1.42	1.51	0.82	0.71	1.29	1.38
Two damages	Middle layer (ply4–ply5)	1.47	1.52	0.83	0.62	1.31	1.44
Three damages	Boundary layer (ply1–ply2)	1.34	1.53	0.78	0.71	1.23	1.27
Three damages	Middle layer (ply4–ply5)	1.29	1.52	0.83	0.70	1.52	1.44

**Table 5:** Metric ratios of curvature mode change rate method relative to surface smoothing algorithm (Table 3/Table 2).

Case	Curvature Indicator	Delamination Location	***R***_PBR_ (Table 3/Table 2)	***R***_Eloc_ (Table 3/Table 2)	***R***_SNR_ (Table 3/Table 2)
Two damages	Modal shape curvature	Boundary layer (ply1–ply2)	1.99	0.52	1.31
Two damages	Modal shape curvature	Middle layer (ply4–ply5)	1.97	0.50	1.36
Two damages	ULS curvature	Boundary layer (ply1–ply2)	1.87	0.60	1.22
Two damages	ULS curvature	Middle layer (ply4–ply5)	1.90	0.68	1.24
Three damages	Modal shape curvature	Boundary layer (ply1–ply2)	1.75	0.62	1.26
Three damages	Modal shape curvature	Middle layer (ply4–ply5)	1.78	0.67	1.30
Three damages	ULS curvature	Boundary layer (ply1–ply2)	1.54	0.68	1.23
Three damages	ULS curvature	Middle layer (ply4–ply5)	1.51	0.79	1.36

**Table 7:** Experimental quantitative metrics for two baseline-free algorithms (modal shape curvature only).

Case	Algorithm	PBR	Eloc/mm	SNR/dB
One delamination	Surface smoothing algorithm	10.96	8.65	12.45
Two delaminations	Surface smoothing algorithm	7.72	10.41	8.05
One delamination	Curvature mode change rate method	12.56	6.46	14.13
Two delaminations	Curvature mode change rate method	9.32	8.34	9.98

**Table 8:** Quantitative comparison between curvature mode change rate method and surface smoothing algorithm based on experimental data (M1 and M2 respectively represent surface smoothing method and curvature mode change rate method).

Case	***R***_PBR_ (M2/M1)	***R***_Eloc_ (M2/M1)	***R***_SNR_ (M2/M1)
One delamination	1.15	0.75	1.14
Two delaminations	1.21	0.80	1.24

## Data Availability

The original contributions presented in this study are included in the article. Further inquiries can be directed to the corresponding author.

## Abbreviations

The following abbreviations are used in this manuscript:

ULS	Uniform Load Surface
PBR	Peak to Background Ratio
SNR	Signal to Noise Ratio
MAC	Modal Assurance Criterion
